# Ultrasmall Hollow Covalent Triazine Framework Nanosphere/Aramid Nanofiber Composite Separator for High‐Energy Lithium Metal Batteries

**DOI:** 10.1002/advs.202510757

**Published:** 2025-08-11

**Authors:** Yufei Yang, Kaveesha Ihala Kodippili, Yun Wang, Jintumol Mathew, Jie Chen, Yi Chen, Xingyan Zeng, Xuyang Wang, Xingping Zhou, Petr Král, Hui Nie, Xiaolin Xie

**Affiliations:** ^1^ Key Laboratory of Material Chemistry for Energy Conversion and Storage Ministry of Education School of Chemistry and Chemical Engineering Huazhong University of Science and Technology 1037 Luoyu Road, Hongshan District Wuhan 430074 P. R. China; ^2^ Department of Chemistry University of Illinois at Chicago Chicago IL 60607 USA; ^3^ Department of Physics Pharmaceutical Sciences and Chemical Engineering University of Illinois at Chicago Chicago IL 60607 USA

**Keywords:** interpenetrative ion pathways, lithium metal batteries, separator, uniform ion migration

## Abstract

Sluggish and uneven mass transport in separators significantly accelerates lithium metal batteries (LMBs) degradation. Here, via scaffold‐coating synergistic strategy, composite separator integrating heat‐resistant, polar aramid nanofiber (ANF) porous scaffold with ultrasmall hollow covalent triazine framework nanosphere (SCTF) coating is fabricated to boost the cycling performance of LMBs across diverse operating conditions. The highly porous ANF layer serves as robust electrolyte reservoir, while the SCTF layer functions as efficient ion redistributor leveraging its extensive interconnected pathways present within staggered layers of intrinsic nanopores with intra‐ and interparticle open spaces. To highlight the importance of mesoscale structure of coatings, the ion transport uniformity and kinetics of SCTF/ANF separator are compared with those of nanosheet‐like CTF and large hollow CTF nanosphere coated separators by time‐of‐flight secondary ion mass spectrometry and molecular dynamics simulations. Owing to its exceptional ionic conductivity (1.41 mS cm^−1^) and Li^+^ transference number (0.79), the SCTF/ANF separator endows the Li//LiFePO_4_ cell with capacity retention of 72% after 3000 cycles at 5 C. The practical viability of this separator is demonstrated by its stable cycling performances in high‐rate, high‐voltage, and high‐temperature LMBs, along with pouch cells. This work highlights the scaffold‐coating synergy and mesoscale coating design of separators for high‐performance LMBs.

## Graphical abstract

In this work, composite separator using high‐strength and polar aramid nanofiber (ANF) is designed as scaffold and ultrasmall hollow covalent triazine framework (CTF) as coating material. This separator demonstrates outstanding performance in high‐rate, high‐voltage, and high‐temperature lithium metal batteries, along with pouch cells.

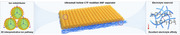

## 1 Introduction

Metallic lithium (Li) has been considered one of the most promising materials for anodes in the next generation of high‐energy‐density rechargeable batteries. This is due to its low redox potential of −3.04 V, low weight density of 0.53 g cm^−3^, and high specific energy density of 3860 mAh g^−1^.^[^ ^1^, ^2^ ^]^ However, the practical adoption of the Li anode is largely hindered by the uncontrollable growth of dendrites during the Li plating/stripping process, leading to poor cycling performance and low Coulombic efficiency (CE) of the batteries.^[^ ^3^, ^4^ ^]^ Continuously growing lithium dendrites on the anode could penetrate the separator, resulting in short circuits and serious thermal runaway.^[^ ^5^, ^6^ ^]^ These challenges have been addressed in numerous studies focusing on the structural engineering of electrodes and electrolytes, such as artificial coating layers for anodes,^[^ ^7^ ^]^ additives for liquid electrolytes,^[^ ^8^ ^]^ and solid‐state electrolytes.^[^ ^9^, ^10^ ^]^

Though the separator is not an active component of the cell, its structure and chemistry, as well as its interaction with the electrolytes, have a considerable impact on ion transport and safety of high‐performance LMBs.^[^ ^11^ ^]^ Fast and homogeneous Li^+^ transport through the electrolyte‐filled porous separator can efficiently relieve the Li^+^ concentration polarization, delay the nucleation of Li dendrites and enable homogeneous lithium plating/stripping, thereby stabilizing the cycling of LMBs.^[^ ^12^, ^13^, ^14^ ^]^ The rate and homogeneity of Li^+^ transport can be largely determined by the chemical compositions and pore structures of the separators. Therefore, regulating the ion transport within the separator is crucial for achieving rapid and uniform Li^+^ flux.

Composite separator, engineered with a mechanically stable porous polymeric scaffold and a surface functional layer, offers a facile approach to enhance the ion transport rate, selectivity and homogeneity in battery.^[^ ^15^, ^16^ ^]^ In most cases, the commercialized polyolefin porous membranes have been exploited as the scaffold due to their high mechanical strength, excellent chemical and electrochemical stability.^[^ ^17^ ^]^ However, the ionic conductivities of the electrolytes within these largely hydrophobic polyolefin membranes are only 5–20% of those in bulk liquid electrolytes,^[^ ^18^ ^]^ where Li^+^ transference number ($t_{Li^{+}}$) = 0.2‐0.4.^[^ ^19^ ^]^ The incomplete and uneven wetting of polyolefin scaffolds causes heterogeneous Li plating and the formation of dendrites. Moreover, fast‐charging under high currents can generate excessive heat and uneven temperature distribution in the battery, increasing the risk of thermal runaway.^[^ ^14^ ^]^ However, composite separators based on polyolefin porous scaffolds always feature an unsatisfied thermal stability, thus raising a critical safety concern of the LMBs.^[^ ^20^ ^]^ Therefore, composite separators built on high‐performance and polar polymeric scaffolds are in urgent need.

Until now, porous organic polymers (POPs), with their modular covalent networks, high surface areas, and tunable nanopores, have emerged as advanced surface modification materials to boost the ion transport performances of composite separators by size confinement and/or electrostatic interactions.^[^ ^21^, ^22^ ^]^ As a typical type of POPs, covalent triazine frameworks (CTFs) with abundant polar and electron‐withdrawing triazine linking units show great promise in promoting fast and selective Li^+^ transport for separators.^[^ ^23^ ^]^ While CTFs with diverse molecular compositions have been exploited as active coating materials to regulate the migration of Li^+^ in LMBs,^[^ ^24^ ^]^ the mesoscale structure of the CTF coating layer in separator depending on the morphology and thus stacking of CTFs was largely ignored. Most synthetic methods for CTFs usually lead to bulky materials or irregular morphologies.^[^ ^25^, ^26^ ^]^ Surface coatings using these bulky materials result in thick layers and nonuniform interparticle pores, which lengthen the ion transport path and sacrifice the homogeneity of Li^+^ flux.^[^ ^27^ ^]^ Meanwhile, it dramatically affects the exposure of active sites and surface areas of the coating layers, which also determine the ion transport behaviors. Additionally, the ion transport properties through the separators, for example, uniformity, can only be indirectly reflected by the electrochemical performance of cells. Therefore, it is crucial to develop high quality characterization techniques and find appropriate models to observe and properly describe the ion transport through modification layers with different mesoscale structures.

**Figure 1:**
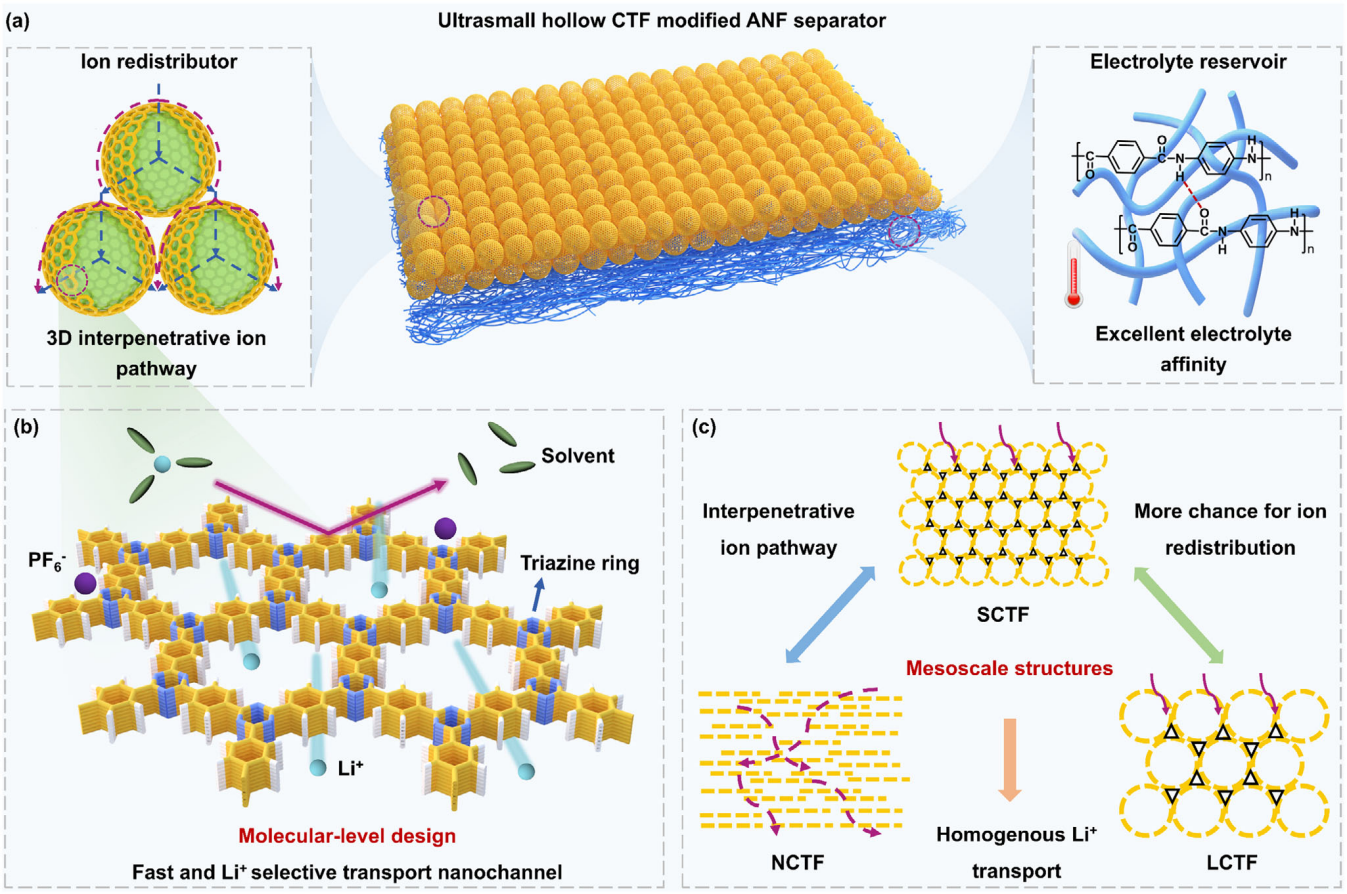
Transport properties of the CTF coating layers with different mesoscale structures. a) Schematic illustration of SCTF/ANF separator. b) CTF with electron‐withdrawing triazine linking units interacts with PF_6_^−^ and accelerates Li^+^ desolvation. c) Schematic illustration of the ion flows in NCTF, LCTF, and SCTF coating layers.

Herein, via a scaffold‐coating synergistic strategy, a composite separator integrating thermally stable, highly polar aramid nanofiber (ANF) porous layer with ultrasmall hollow covalent triazine framework (SCTF) coating layer (SCTF/ANF) is fabricated via a simple two‐step film‐casting process combined with a phase inversion approach (**Figure** **1**a). Thanks to the abundant hydrophilic amide groups, high strength and high‐temperature resistance, the ANF layer acts as robust electrolyte reservoir and ensures excellent electrolyte affinity for separator. Meanwhile, the abundant electron‐withdrawing triazine linking units in SCTF interacts with PF_6_^−^ and accelerates Li^+^ desolvation in the electrolyte, leading to selective and fast Li^+^ transport (Figure 1b).^[^ ^28^ ^]^ The hollow morphology and ultrasmall size of SCTF enables the formation of ultra‐uniform and thin coating layer and provides 3D interpenetrative ion pathways for efficient ion redistribution. Through such separator design, efficient acceleration and modulation of ion transport can be achieved for stable Li metal anodes. To further illustrate the importance of mesoscale structure of CTF coating layers for separators, three types of CTFs are synthesized: 1) nanosheet‐like CTF (NCTF), 2) large hollow CTF nanosphere (LCTF), and 3) SCTF. Through time‐of‐flight secondary ion mass spectrometry and molecular dynamics simulations, the distributions of ion fluxes through coating layers with different mesoscale structures are visualized and quantified. Compared to NCTF and LCTF layers, the stacked hollow nanospheres with thin shells and large open space present in SCTF layer could provide more homogeneous ion transport along largely shortened and more interpenetrative ion pathways (Figure 1c). Thus, the Li//LiFePO_4_ (LFP) cell employing the SCTF/ANF separator demonstrates superior cycling stability, delivering an initial capacity of 120.0 mAh g^−1^ at 5 C with 72% capacity retention after 3000 cycles. Impressively, the SCTF/ANF separator also exhibits highly competitive overall performance in high‐voltage LMBs, high‐temperature LMBs, and pouch cells with high‐mass‐loading LFP cathode (32.3 mg cm^−2^).

## 2 Results and Discussion

### 2.1 Design of CTF Modified ANF Separators

**Figure 2:**
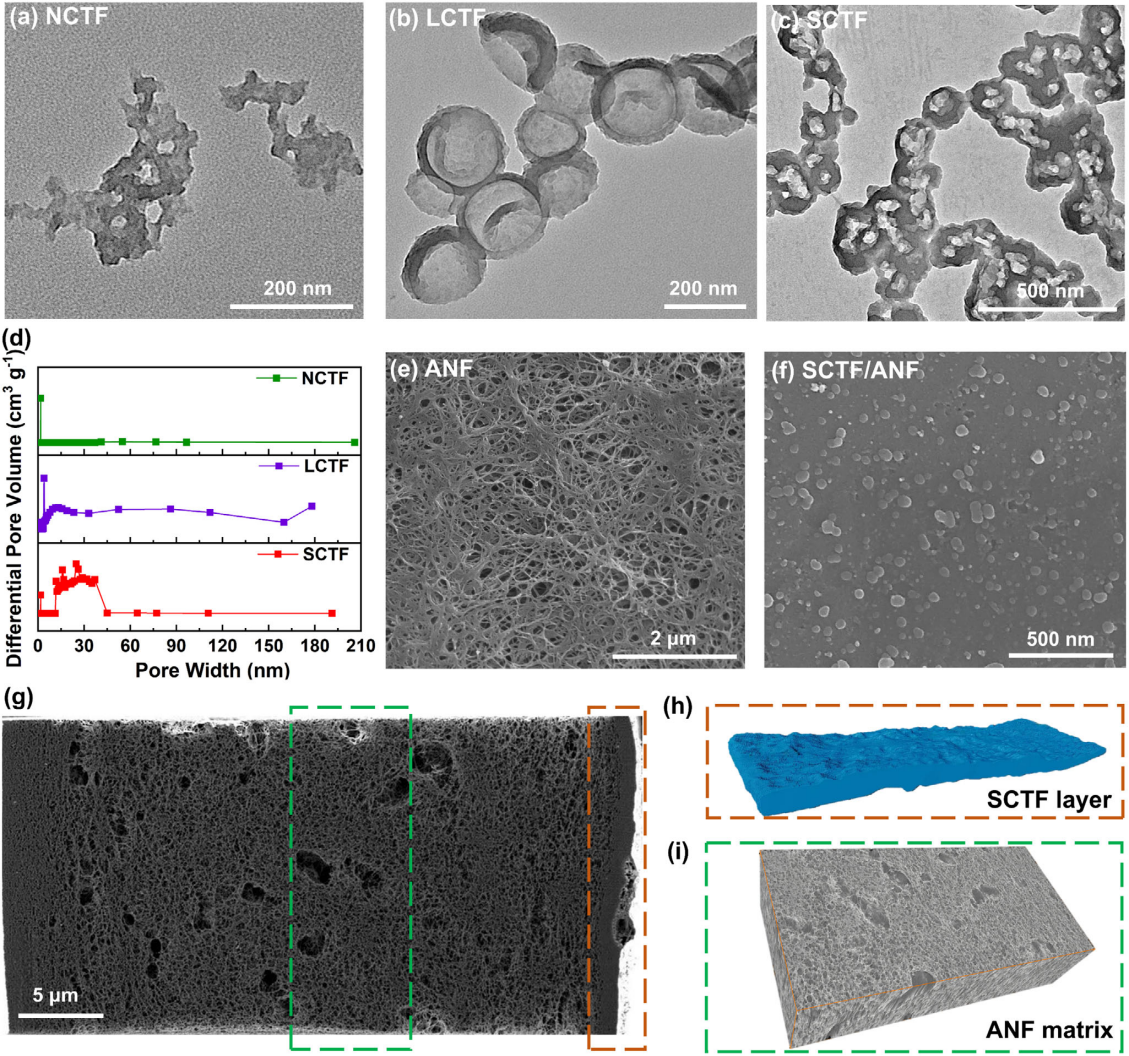
Characterization of CTFs and CTF modified ANF separators. Field emission transmission electron microscopy (FE‐TEM) images of a) NCTF, b) LCTF, and c) SCTF. d) Pore size distributions of NCTF, LCTF and SCTF. Top‐view FE‐SEM images of e) ANF porous membrane and f) SCTF side of SCTF/ANF separator. g) Cross‐sectional FIB‐SEM image of SCTF/ANF separator. h) Segmentation of the 3D FIB tomography of SCTF layer. i) 3D reconstruction of the ANF scaffold with dimensions of 11.9 × 23.9 × 5.9 µm^3^ by integrating a series of FIB‐SEM slices.

NCTF, LCTF, and SCTF were synthesized by condensation reaction of aldehyde and amidine under mild conditions. The one‐pot method^[^ ^29^ ^]^ was utilized to synthesize NCTF with a thickness of 30 ± 7 nm (**Figure** **2**a; Figure S1, Supporting Information). A modified template method was developed for LCTF and SCTF (Figure S2, Supporting Information),^[^ ^26^ ^]^ where CTF was grown on the surface of SiO_2_ templates (average sizes of ≈180 and 30 nm) to generate SiO_2_@CTF core‐shell structures (Figure S3, Supporting Information). Afterward, the SiO_2_ templates were etched to obtain the CTF hollow nanospheres with different cavity volumes (Figure 2b,c). With a one‐step addition of reactants, CTF could easily grow on the surface of SiO_2_ template with a diameter of ≈180 nm. However, CTF tends to grow in solution, generating bulky materials, as well as on the surface of 30 nm SiO_2_ nanoparticles used as the template. To overcome this challenge, the controlled addition of aldehyde monomer through a peristaltic pump was exploited at a feed rate of 0.05 mL min^−1^ to regulate the nucleation process and slow down the growth rate of CTF, ensuring the generation of core‐shell structures. The average shell thickness and the cavity size are ≈15 and 180 nm for LCTF, and 4 and 30 nm for SCTF, respectively. Due to the thin shells of LCTF and SCTF, some nanospheres may collapse, resulting in irregular spherical structures. The pore size distribution of LCTF and SCTF evaluated by the N_2_ sorption isotherm at 77 K further confirms their hollow morphology (Figure 2d). The LCTF and SCTF show macropores or mesopores structures, which derives from the cavity of the hollow nanospheres or voids between the stacked nanospheres. Meanwhile, the micropores from the NCTF and CTF shells of LCTF and SCTF are also observed. For the synthesis of hollow CTF nanospheres, controlled addition of aldehyde monomer is employed to generate low‐density nuclei, thereby facilitating crystal growth. The enhanced crystallinity of LCTF and SCTF than NCTF is suggested by the intensity of exposed peaks in the powder X‐ray diffraction (PXRD) patterns (Figure S4, Supporting Information).^[^ ^26^ ^]^ Characteristic vibrations of the triazine units at 1518 and 1354 cm^−1^ are observed in the Fourier transform infrared (FT‐IR) spectra of NCTF, LCTF, and SCTF. Meanwhile, the characteristic peak of Si─O (1078 cm^−1^) disappears in LCTF and SCTF. Furthermore, from the X‐ray photoelectron spectroscopy (XPS) measurement, the deconvolution of N 1s peaks around 398.8 eV from NCTF, LCTF and SCTF layers were attributed to pyridine nitrogen (C═N─C) (Figure S5, Supporting Information), confirming the formation of triazine framework.

### 2.2 Characterizations of CTF Modified ANF Separators

Through the two‐step film‐casting process combined with a phase inversion approach developed in our group,^[^ ^30^ ^]^ large‐scale fabrication of the NCTF coated ANF separator (NCTF/ANF), LCTF coated ANF separator (LCTF/ANF), and SCTF/ANF separator can be achieved (Figure S6, Supporting Information). A dark red CTF layer on top of the beige ANF membrane is observed for the three types of separators (Figure S7, Supporting Information). The top‐view images of field emission scanning electron microscopy (FE‐SEM) indicate that the ANF separator has fiber structures and abundant pores (Figure 2e). And spherical or flaky CTFs are evenly distributed on the surface of the CTF/ANF composite separators (Figure 2f; Figure S8, Supporting Information). The cross‐sectional structure of the separator was examined by focused ion beam scanning electron microscopy (FIB‐SEM). The SCTF/ANF separator comprises a tightly bonded CTF coating layer and ANF scaffold (Figure 2g) with thicknesses of ≈400 nm and 40 µm, respectively. The firm attachment of the CTF layer on ANF scaffold is evidenced by the absence of cracks after repeated bending, which may be originated from the hydrogen bonding between the two layers (Figures S9,S10, Supporting Information). 3D reconstruction of the ANF scaffold (11.9 × 23.9 × 5.9 µm^3^) by FIB‐SEM shows the interconnected porous structure (Figure 2i), which is essential for ion transport. To be able to compare the effect of mesoscale structures of the coating layer on the properties of the separator, the thickness of NCTF, LCTF, and SCTF modification layers was fixed at the same value by using the same slurry concentration and coating procedures. Their porosity was obtained by measuring the weight of separators before and after immersion in *n*‐butanol, where the tortuosity was calculated from the electrochemical impedance spectroscopy (EIS) experiments (Figure S11 and Table S1, Supporting Information).^[^ ^31^ ^]^ The pore size distributions and surface areas of the obtained NCTF/ANF, LCTF/ANF, and SCTF/ANF separators are shown in Figure S12 and Table S1 (Supporting Information). These three separators have surface areas of 223.73, 198.06, and 213.14 m^2^ g^−1^, respectively. The NCTF coating layer has the largest surface area due to the smallest average pore size. Compared with the NCTF/ANF separator, LCTF/ANF and SCTF/ANF separators have slightly higher porosity and lower tortuosity, indicating that coating layers with CTF hollow nanospheres have better connected porous structure.

The mechanical properties of CTF/ANF separators are similar to that of pristine ANF, with strength and strain at failure of ≈25 MPa and 6.5%, respectively, which are comparable to those of Celgard 2400 in the transverse direction (Figure S13, Supporting Information). The effect of the mesoscale structure of coating layers on the physicochemical properties of the separators was investigated. The contact angles of pristine polyethylene (PE), ANF, NCTF/ANF, LCTF/ANF, and SCTF/ANF separators with liquid electrolytes are 44.3, 32.3, 29.3, 28.0, and 26.1°, respectively, manifesting the improved electrolyte wettability of CTF coated ANF separators (Figure S14, Supporting Information). The outstanding electrolyte wettability is primarily attributed to the abundant nanopores and interaction between the polar triazine rings of the CTF skeleton and electrolytes.^[^ ^32^ ^]^ Except for electrolyte wettability, the LCTF/ANF and SCTF/ANF separators feature higher electrolyte uptake and retention (Figure S15, Supporting Information).^[^ ^33^ ^]^ The substantially enhanced electrolyte wettability and uptake improve the homogeneity of Li^+^ flux. The linear sweep voltammetry (LSV) curves show that PE, ANF, NCTF/ANF, LCTF/ANF, and SCTF/ANF separators can operate in a stable manner at a voltage range of U = 2.5−4.5 V (Figure S16, Supporting Information), suggesting that these composite separators are suitable for LMBs.

### 2.3 Quantification of Ion Fluxes Through Separators

**Figure 3:**
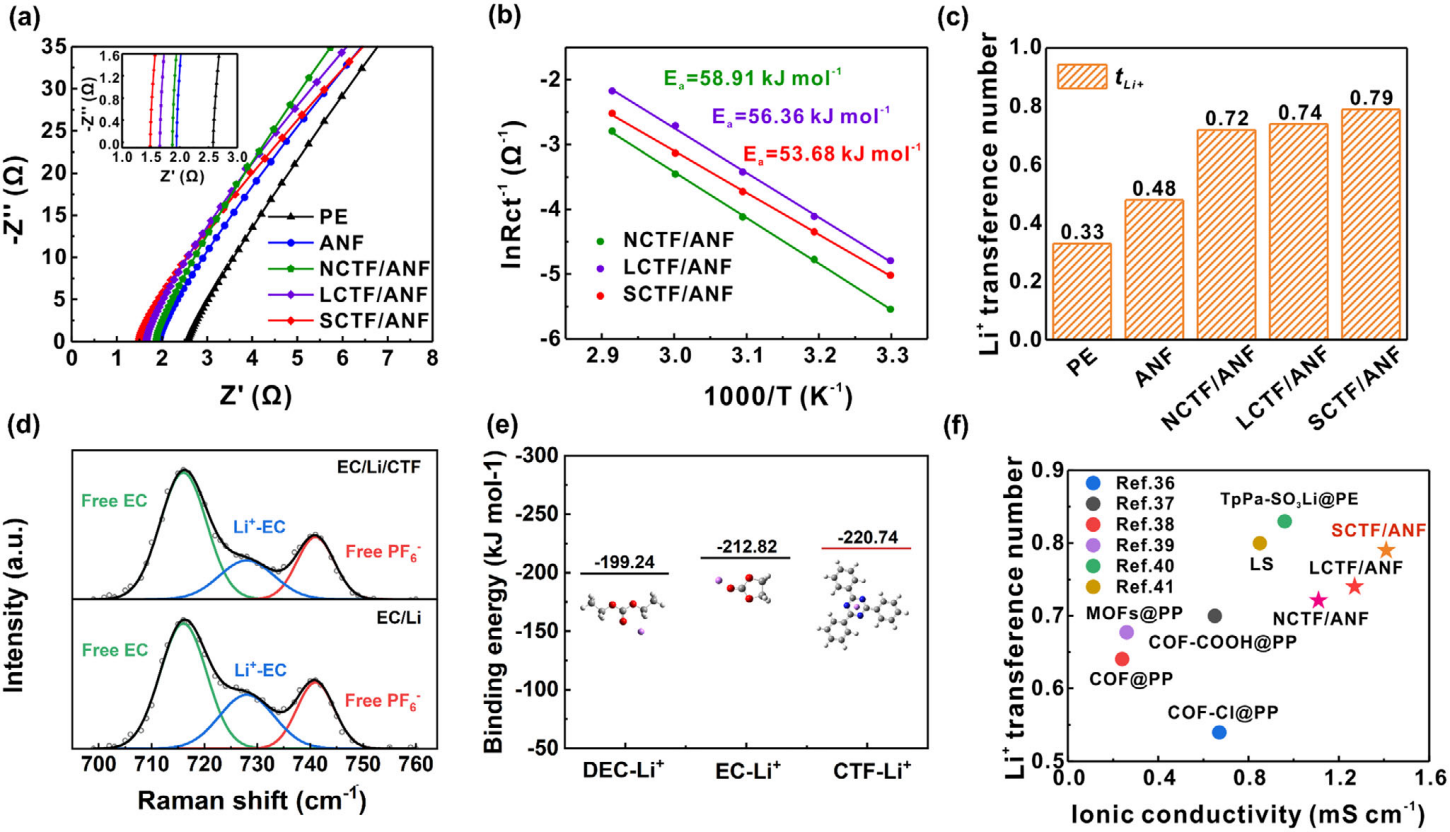
Characterization of the CTF modified ANF separator. a) EIS plots of the PE, ANF, NCTF/ANF, LCTF/ANF, and SCTF/ANF separators. The inset shows enlarged EIS plots in the high‐frequency region. b) The calculation of *E_a_* from Nyquist plots. c) $t_{Li^{+}}$ of PE, ANF, NCTF/ANF, LCTF/ANF, and SCTF/ANF separators. d) Raman spectra of EC/Li and EC/Li/CTF. e) Gibbs free energy for the DEC‐Li^+^, EC‐Li^+^ and CTF‐Li^+^ and their 3D structures. f) Comparison of ionic conductivity and $t_{Li^{+}}$ of CTF modified ANF separators with other separators in literature.^[^ ^36^, ^37^, ^38^, ^39^, ^40^, ^41^ ^]^

We measured the ionic conductivities of these composite separators to clarify the effect of different coating layers on their electrochemical performance. Using the EIS measurement, the PE, ANF, NCTF/ANF, LCTF/ANF, and SCTF/ANF separators show ionic conductivities of 0.19, 1.05, 1.11, 1.27, and 1.41 mS cm^−1^, respectively, as illustrated in **Figure** **3**a and Table S2 (Supporting Information). To demonstrate the desolvation effect of the CTF layers, the activation energy (*E_a_*) of ion desolvation was calculated according to the Arrhenius equation, 1/*R_ct_* = A *exp*(− *E_a_*/RT),^[^ ^34^ ^]^ where *R_ct_*, A, *E*_a_, R, and T represent the charge transfer resistance, frequency factor, activation energy, ideal gas constant, and absolute temperature, respectively. As shown in Figures 3b and S17 (Supporting Information), the SCTF/ANF separator has the lowest activation energy of *E_a_* = 53.68 kJ mol^−1^, suggesting the desolvation effect of triazine groups for Li^+^ and implying rapid ion transfer kinetics. To evaluate the selective transport ability of Li^+^ for different separators, the $t_{Li^{+}}$ was measured by the Bruce‐Vincent method.^[^ ^35^ ^]^ The chronoamperometry profiles of separators assembled Li//Li symmetric cells are shown in Figures S18–S22 (Supporting Information), revealing the PE, ANF, NCTF/ANF, LCTF/ANF, and SCTF/ANF separators have $t_{Li^{+}}$ = 0.33, 0.48, 0.72, 0.74, and 0.79, respectively (Figure 3c). Raman spectroscopic measurements of ethylene carbonate (EC)/diethyl carbonate (DEC) containing 1 m LiPF_6_ (EC/Li) and CTF in EC/Li electrolyte (EC/Li/CTF) were performed to elucidate the interactions between CTF, LiPF_6_ and solvent molecules. As shown in Figure 3d, EC/Li and EC/Li/CTF show three main peaks at 716, 728, and 742 cm^−1^, which are attributed to the free EC, Li‐EC complex and free PF_6_^−^ anion, respectively. The intensity ratios of PF_6_^−^ to EC (free PF_6_^−^/free EC) for the EC/Li and EC/Li/CTF are 0.42 and 0.39, respectively, indicating that the PF_6_^−^ anions are partially attached to the CTF. Furthermore, the intensity ratios of free EC to Li‐EC (free EC/Li‐EC) for the EC/Li and EC/Li/CTF are 1.97 and 2.76, respectively, suggesting that Li^+^ is solvated by fewer EC molecules with the existence of CTF with polar triazine rings. The immobilized PF_6_^−^ anions on the CTF and solvation shell with a smaller number of EC molecules enhance Li⁺ mobility in battery. The binding energy between Li^+^, solvents and CTF are further studied using first‐principles density functional theory (DFT) calculations. As shown in Figure 3e, the binding energy of Li^+^ and CTF is −220.74 kJ mol^−1^. Their interaction is stronger than that of EC (−212.82 kJ mol^−1^), and DEC (−199.24 kJ mol^−1^), implying that the CTF can promote the desolvation process of Li^+^. Therefore, the synergistic effect of the desolvation and electrostatic interaction between anion and CTF enable both high $t_{Li^{+}}$ and ionic conductivity. The advantages of the SCTF/ANF separator become evident when compared with other related systems. Figure 3f compares the performance of POPs‐modified separators in literature,^[^ ^36^, ^37^, ^38^, ^39^, ^40^, ^41^ ^]^ NCTF/ANF, LCTF/ANF, and SCTF/ANF separators. The SCTF/ANF separator exhibits one of the highest ionic conductivities of 1.41 mS cm^−1^ and $t_{Li^{+}}$ of 0.79, while achieves a good balance between the two benefiting the relief of concentration polarization.

To better understand the effect of mesoscale structures of the coating layers on the ion transport of the composite separators, we describe these systems by a simplified quasi‐continuum modelling.^[^ ^42^ ^]^ The coating layer of each separator can be viewed as a porous network of interconnected pores consisting of a composite of CTF particles and polymeric binder filled with liquid electrolytes. The effective electrolytic conductivity of this porous layer can be obtained from

(1) $$\sigma_{eff}=\varepsilon^{\alpha }\sigma_{0},\alpha ≅1.5$$

where σ_0_ is the bulk ionic conductivity of the electrolyte, ε is the void volume fraction of the separator filled with electrolyte, and α is the Bruggeman exponent. Typically, α = 1.5 holds for spherical particles, while α > 3 holds for anisotropic particles.^[^ ^42^ ^]^ Ions passing through porous networks of particles present in lamellar and flaky materials travel along increasingly tortuous paths, resulting in a significantly increased α and reduced ionic conductivity of NCTF. In addition, compared to the NCTF flat surface, the LCTF and SCTF surfaces with spherical corrugation have ≈1.57 times larger areas in contact with the liquid electrolyte (Figure S23, Supporting Information). Therefore, ions can enter through more pores in the LCTF and SCTF spheres‐covered surfaces.

### 2.4 Molecular Dynamics Simulation of Ion Flows

**Figure 4:**
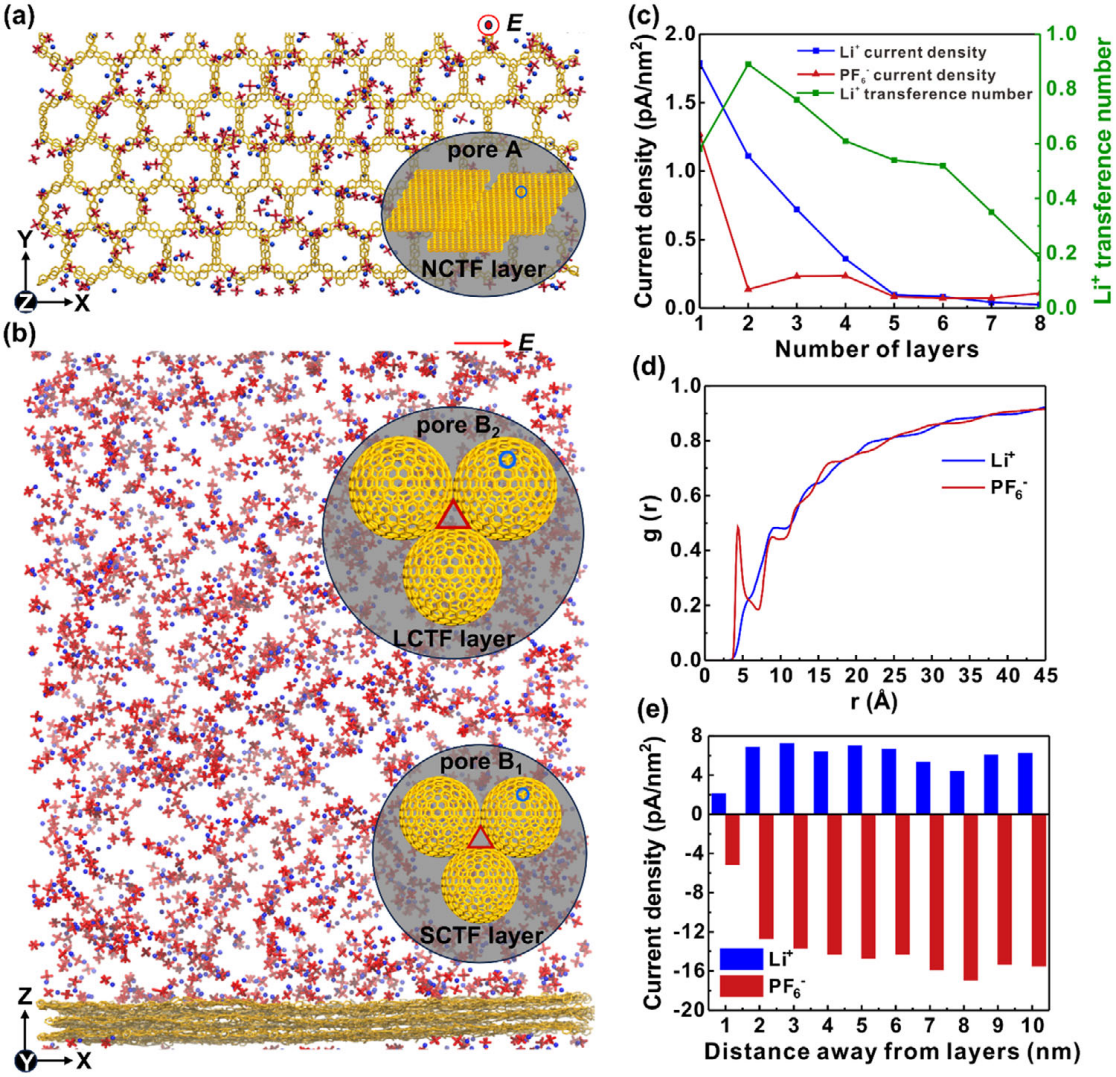
Atomistic MD simulations of electric‐field‐driven Li^+^ and PF_6_^−^ transport through CTF coating layers. a) Ion flow through pores A in CTF layers (top view). b) Ion flow through pore B_1_ and pore B_2_ (front view). c) Current density of Li^+^ and PF_6_^−^ (the ions move in opposite directions) for different numbers of CTF layers (stacked on top of each other) in pore A and corresponding $t_{Li^{+}}$. d) Distribution of ions with distance from the CTF layers. e) Current densities of ions at different distances from the CTF surface. Regions adjacent to the layers can mimic transport through pore B_1_ (less solvent), while regions further away from the layers can mimic transport through pore B_2_ (more solvent).

To better understand the electric‐field‐driven ion transport through the experimental separators, we model these systems by molecular dynamics (MD) simulations. The coating layers of separators possess two main types of pores for ion transport: A) nanopores within the CTF sheets and hollow nanospheres (1.2 nm, pore A), and B) nanopores (open space or voids) in and between the stacked CTF hollow nanospheres (pore B) (**Figure** **4**). Under ideal conditions, the CTF hollow nanospheres in the coating layers are closely packed to form a network of octahedral and tetrahedral interparticle voids with sizes proportional to nanosphere sizes.^[^ ^43^ ^]^ In the real systems, some channels could also form between (orthogonal to) individual sheet layered either in a planar (sheets) or spherical way (nanospheres). We simulate the electric‐field‐driven transport of ions passing through nanopores within different numbers of CTF sheets (pore A) (Figure 4a) and ions passing at different distances above the surfaces of CTF sheets (pore B) (Figure 4b). First, we simulate electric‐field‐driven transport through pores A present within (1–8) stacked CTF layers that can correspond to different regions within NCTF, LCTF and SCTF. The systems are formed by flat CTF layers (16.1 × 17.8 nm^2^) solvated in a LiPF_6_ (1 m) solution of ethylene and diethyl carbonate (1:1 volume ratio), matching the experiments. All systems were simulated in an electric field of *E*  =  2.17 mV/nm until the transport reached a steady state flow. In pore A, the field is applied across the layers (z direction), while in pore B it is parallel to them (x direction). The numbers of cations and anions passing through or along the CTF layers were counted by averaging over the trajectory after the systems reached steady state (through 160 and along 280 ns). As shown in Figure 4c and Tables S3,S4 (Supporting Information), the ion transport rates become dramatically decreased (1/n, n: number of layers) when the number of CTF layers grows, so the flow becomes very small for more than 3–5 layers. The $t_{Li^{+}}$ also drop from the maximum of ≈0.89 at two layers to ≈0.30 for 5 layers. Collectively, these findings establish that minimal layer thickness is essential to achieve simultaneous fast and selective ion conduction.

Next, we study ion transport through pores B (nanopores B_1_ and B_2_ are present between 3 neighboring small and large hollow nanospheres, respectively). Since the actual sizes of these inter‐ or intraparticle pores depend on the sizes of nanoparticles, we calculate the current densities at different distances from the CTF sheets (Figure 4b). We start by calculating the equilibrium distributions of both ions above the CTF sheets (Figure 4d). These distributions reveal that PF_6_^−^ anions are partly attached to the CTF sheets (peak at 5 Å) due to electron‐withdrawing triazine rings of the CTF surface.^[^ ^44^ ^]^ This surface charge is compensated (screened) by the enhanced presence of Li^+^ within the next 5–7 Å away from the surface, thus creating an electric double layer. The current densities, calculated next (Figure 4e), show that the flow of both ions is reduced close to the CTF surface, but the $t_{Li^{+}}$ ≈ 0.35–0.4, are somewhat elevated (only cations present). At 3–6 nm separations from the surface, the current densities are already stabilized and giving “bulk” current densities and smaller $t_{Li^{+}}$ of ≈0.25–0.32. These values can be considered as the average $t_{Li^{+}}$ for the B_1_ pore (regions adjacent to the layers) and B_2_ pore (regions further away from the layers) present in the CTF lattices (Table S3, Supporting Information). These results reveal that increased size of interparticle pores directly compromises the $t_{Li^{+}}$, highlighting the critical role of pore size regulation in CTF layers.

Ion transport through the CTF layers occurs simultaneously within the A and B pores. Based on above simulation results, the average $t_{Li^{+}}$ and current densities for different CTF systems are estimated. Remarkably, thanks to the spherical geometries, the SCTF and LCTF demonstrate a 57% increase in accessible ion transport surfaces (pore A) compared to the NCTF layer (Figures S23,S24, and Table S5, Supporting Information). Furthermore, the thinnest shells and most compact dimensions of SCTF layer enable unparalleled ion transport selectivity and kinetics.

### 2.5 Visualization of Ion Fluxes Through Separators

To further quantify and visualize the mass transport behavior of the separators including permeability and tortuosity, a setup using thermally activated salt was built and time‐of‐flight secondary ion mass spectrometry (TOF‐SIMS) was exploited to investigate the transient transport of electrolytes through the separators.^[^ ^45^ ^]^ The TOF‐SIMS combines lateral imaging and depth profiling with precise elemental detection to create two‐dimensional maps of the ionic concentration. The transport of the thermally activated eutectic lithium chloride‐potassium chloride mixture with a melting point of 352 °C through the separators was tracked. To fulfill these experiments, the separators should be highly thermally stable. As shown in Figures S25,S26 (Supporting Information), the thermal gravimetric analysis (TGA) measurement reveals that up to ≈94% of their weight are retained at temperatures up to 500 °C for ANF, NCTF/ANF, LCTF/ANF, and SCTF/ANF separators. In addition, ANF, NCTF/ANF, LCTF/ANF, and SCTF/ANF separators show no obvious endothermic peak below 360 °C from differential scanning calorimetry (DSC) curves, illustrating the exceptional thermal stability of separators. The above results suggest that the separators can maintain the structural stability for the transport of thermally activated salt at high temperatures.

**Figure 5:**
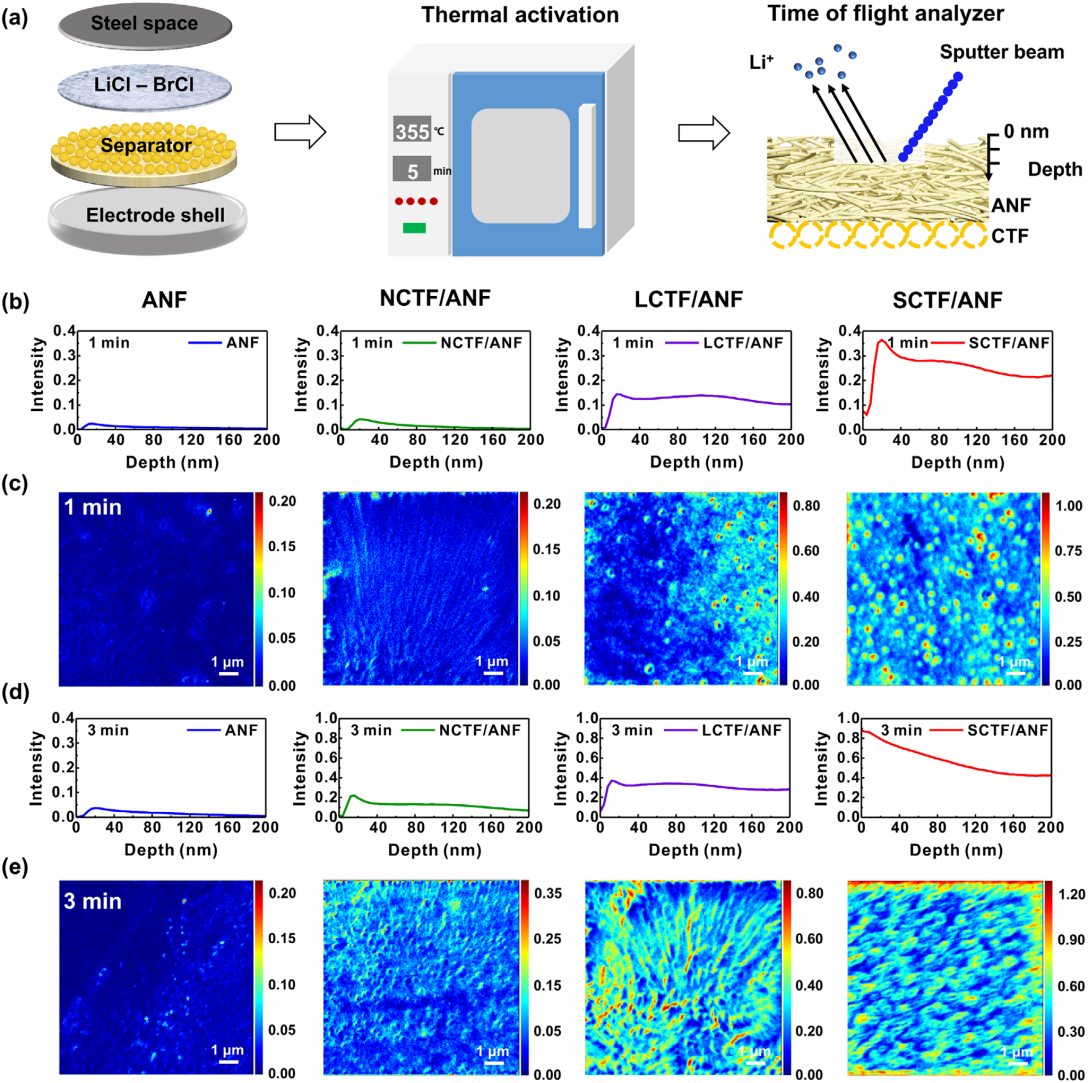
The transient transport of electrolytes through different separators. a) Schematic diagram of the experimental setup for quantifying the mass transport behavior of separators using thermally activated salts and TOF‐SIMS. Li^+^ concentration and their compiled maps across the ANF, NCTF/ANF, LCTF/ANF, and SCTF/ANF separators for the various active times of b, c) 1 min, and d, e) 3 min by TOF‐SIMS depth profiling. b, d) Plot of Li^+^ concentration versus depth. c, e) Top‐view images of Li^+^ concentration. The unit of intensity is counts per extraction (cts/ex). One extraction corresponds to one pulse of the TOF or equivalently, one pixel in the FIB scan.

A schematic of the experimental setup is shown in **Figure** **5**a. The cells were fabricated by sandwiching the separator and salt tablet between the stainless‐steel spacer and the positive electrode shell. Then, the cells were placed in the muffle furnace and held at a constant temperature of 355 °C for a certain period to activate the salt and allow it to be transported through the separator. After a set of duration, the cells were removed from the muffle furnace and quickly subjected to room temperature to freeze the transport. These separators were then subjected to TOF‐SIMS measurements for depth profiles of element concentration maps (Figure 5b–e; Figure S27, Supporting Information). For comparison, separators of almost the same thickness, and salt tablet of the same weight and diameter are used for experiments. The Li^+^ element concentration on the opposite side of the separator from the salt tablet is investigated at activation times of 1 and 3 min. It is quantified by averaging the concentrations as a function of the distance along the separators. Figures 5b–e and S27 (Supporting Information) show that as the activation time increases, higher concentrations of Li^+^ and K^+^ could be detected at a larger distance from the separator to the salt source. The concentration of Li^+^ across ANF, NCTF/ANF, LCTF/ANF, and SCTF/ANF separators are 0.002, 0.011, 0.055, and 0.848 at the same depth with an activation time of 3 min, respectively. The same trend is observed at activation times of 1 min. A significant increase in the concentration of Li^+^ of the coated separators indicates that the polar CTF modification layer can substantially enhance the ion adsorption and transport driven by gravity and capillary force.^[^ ^46^ ^]^ And the depth profiles of K^+^ share similar trend as that of Li^+^ (Figure S27, Supporting Information). The SCTF/ANF separator enables the fastest ion transport. These results are consistent with the trends of ionic conductivity from EIS measurements.

More excitingly, the Li^+^ distribution in different separators could also be evaluated from the TOF‐SIMS measurements. Here, as the ion transport mainly happens through the packed electrolytes in separator pores, an extremely nonuniform ion distribution of Li^+^ flux through the ANF separator is observed due to its uneven pore distribution (Figure 2e). Although the nanopores of CTF skeleton in coating layer can homogenize the ion flux, the stacking of sheet‐like structures or nanospheres form cracks or nonuniform voids, which also leads to the uneven transport of ions. Thus, building highly interpenetrative ion channels and decreasing the size of voids are the key to offset the negative impact. The CTF hollow nanospheres act as nano‐sponge for liquid electrolyte and provide rich 3D Li^+^ migration pathways. The SCTF layer exhibits the most efficient ion redistribution and uniform pore size, leading to the most homogenous ion transport. All these results reveal that the mesoscale structure of the coating layer significantly affects the ion transport rate, selectivity and homogeneity. According to Sand's time model,^[^ ^47^, ^48^ ^]^ it is expected that the separator with high $\;t_{Li^{+}}$, ionic conductivity and uniform ion distribution is capable of delaying the growth of lithium dendrites and achieves more uniform and stable lithium deposition.^[^ ^49^ ^]^

### 2.6 SCTF/ANF Separator for High‐Energy LMBs

**Figure 6:**
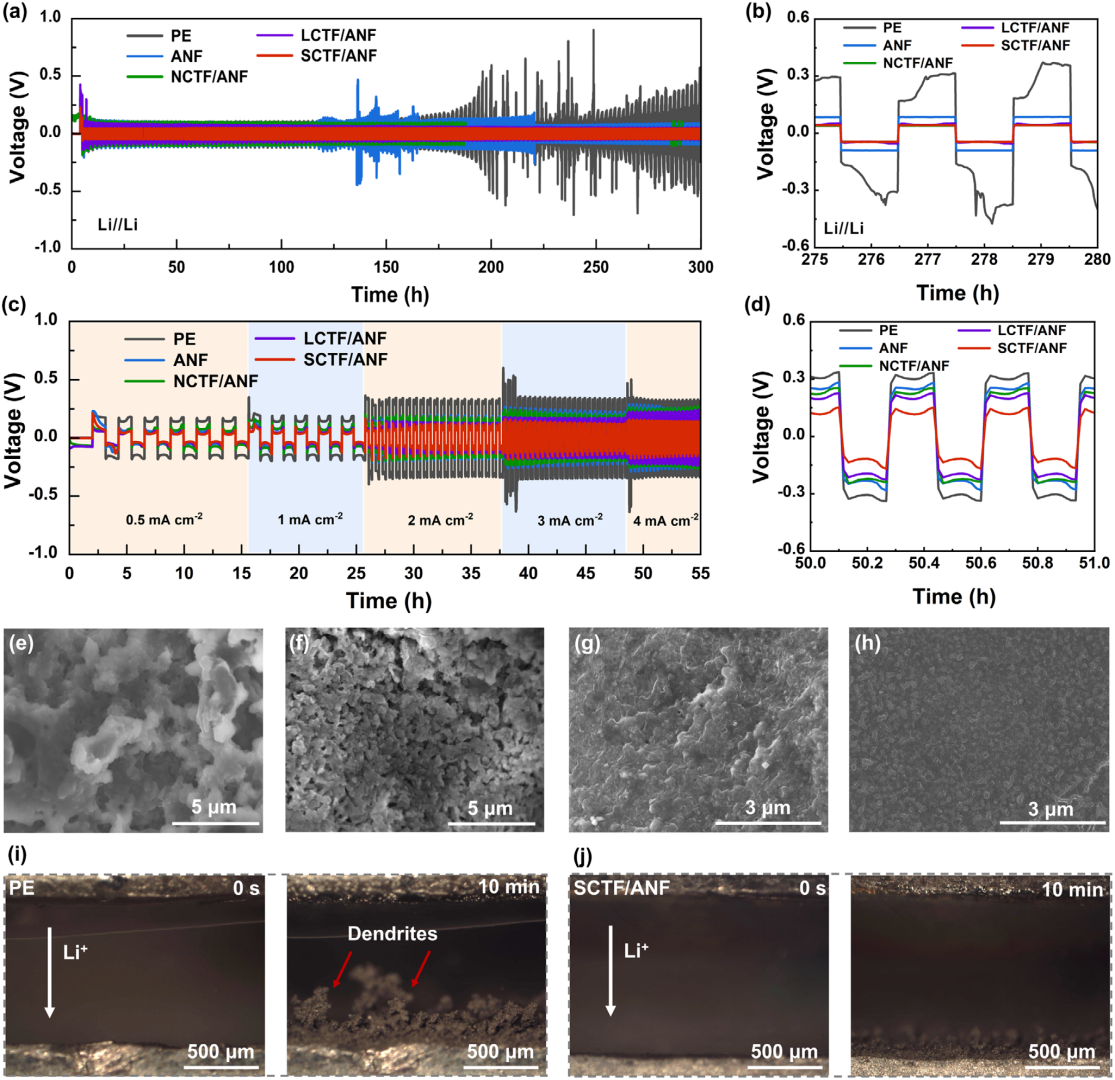
Electrochemical performance of CTF modified ANF separators. a,b) Voltage‐time profiles of Li//Li symmetric cells assembled with PE, ANF, NCTF/ANF, LCTF/ANF, and SCTF/ANF separators at 1 mA cm^−2^ with a capacity of 1 mAh cm^−2^. c,d) Voltage‐time profiles of Li//Li symmetric cells assembled with PE, ANF, NCTF/ANF, LCTF/ANF, and SCTF/ANF separator at current densities from 0.5 to 4 mA cm^−2^ with capacity of 1 mAh cm^−2^. FE‐SEM images of Li metal from Li//Li cells assembled with e) ANF, f) NCTF/ANF, g) LCTF/ANF and h) SCTF/ANF separators after Li plating at a current density of 1 mA cm^−2^ after 100 h. i, j) Morphology of Li in Li//Li symmetrical cells with PE and SCTF/ANF separators after discharging for 10 min at 3 mA cm^−2^ using in situ optical microscopy.

To investigate the ionic transport properties of separators and its impact on the cycling stability of Li anode. First, the CE of the Li||Cu cells with different separators were tested at current densities of 0.5 mA cm^−2^. As shown in Figure S28 (Supporting Information), the cell with SCTF/ANF separator shows a stable and high CE for over 150 cycles, indicating the formation of a stable solid electrolyte interphase (SEI) layer and uniform lithium deposition. To further investigate the lithium deposition behavior of different separators, the long‐term galvanostatic discharging and charging voltage profiles of Li//Li symmetric cells were measured. **Figure** **6**a shows the performance of Li//Li symmetric cells with PE, ANF, NCTF/ANF, LCTF/ANF, and SCTF/ANF separators at a current density of 1 mA cm^−2^ and a fixed capacity of 1 mAh cm^−2^. The overpotential of the symmetric cells assembled with PE and ANF separators gradually increases as early as 110 h and fluctuates dramatically afterward. This results from the rapid electrolyte consumption and lithium dendrite growth caused by side reactions and polarization phenomena in the cells.^[^ ^50^ ^]^ The overpotential of symmetric cells with CTFs modified ANF separators is lower than that of PE and ANF separators. However, the overpotential of the symmetric cell with NCTF/ANF separator decreases suddenly at 180 h, suggesting a short circuit happens in the cell. In contrast, the Li|LCTF/ANF|Li and Li|SCTF/ANF|Li cells cycle over 300 h without apparent voltage fluctuation, illustrating a uniform Li plating/stripping. Note that the SCTF/ANF separator assembled cell has a lower overpotential (Figure 6b), which means the SCTF/ANF separator has the best cycling performance and dendrite inhibition ability. When switching between different current densities from 0.5 to 4 mA cm^−2^ at an area capacity of 1 mAh cm^−2^, the SCTF/ANF separator maintains a polarization profile that is more stable than the others (Figure 6c,d). To further investigate the dendrite‐suppressing effect of the SCTF/ANF separator, the morphology of the lithium plating on Li metal with different separators was monitored at a current density of 1 mA cm^−2^. As shown in Figure S29 (Supporting Information), a large amount of dendritic lithium grows on the Li anode with PE separator. Mossy dendrites are observed on the surface of the Li anode for the ANF and NCTF/ANF separators assembled cells (Figure 6e,f). Dense flaky lithium is observed for the cells with LCTF/ANF and SCTF/ANF separators (Figure 6g,h). The fast and uniform transport of Li^+^ of the SCTF/ANF separator brings about a flat and dense morphology during the lithium deposition process. The chemical composition of SEI layers and separators are further analyzed using XPS. A significantly higher concentration of LiF is detected on the SEI layer of SCTF/ANF separator (Figure S30, Supporting Information), which enhances the interface stability between lithium metal anode and separator because of the high electronic insulation and shear modulus of LiF. According to XPS spectra of NCTF/ANF, LCTF/ANF, and SCTF/ANF separators in Li//Li symmetric cells before and after cycling (Figure S31, Supporting Information), the chemical composition of CTF coating remains substantially unchanged after cycling, demonstrating the good chemical stability of CTF coating during the Li plating/stripping process. To validate the efficiency of SCTF/ANF separator in suppressing dendritic growth, in situ optical microscopy was exploited to investigate the morphology of lithium during plating on the electrodes with PE and SCTF/ANF separators at 3 mA cm^−2^. As shown in Figures 6i,j and S32 (Supporting Information), the electrodes with PE, ANF, NCTF/ANF, LCTF/ANF, and SCTF/ANF separators exhibit smooth surfaces before lithium plating. However, after 600 s, heterogeneous lithium deposition can be observed on the surface of Li anode of cell with PE separator, and loosely structured lithium dendrites grow with a large thickness of ≈300 µm. In sharp contrast, throughout the entire plating process, a compact and flat surface (thickness of ≈77 µm) is observed on the Li anode of cell with SCTF/ANF separator, further confirming the high efficiency of SCTF coating in suppressing dendritic growth. Based on the above result, the effects of coating layers with different mesoscale structures on Li^+^ transport and Li plating behaviors are schematically illustrated in Figure S33 (Supporting Information).

**Figure 7:**
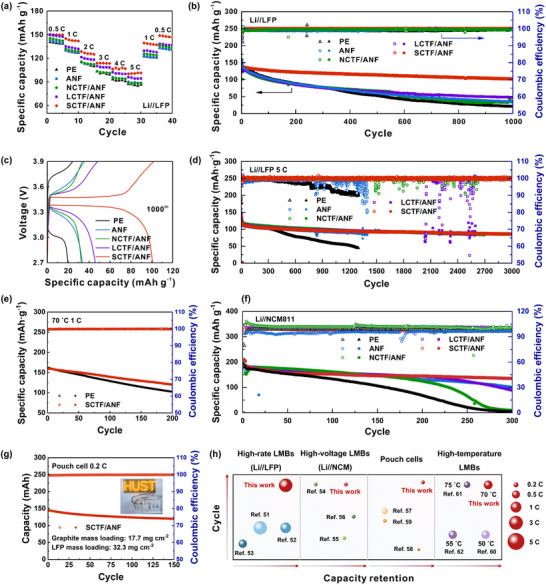
Electrochemical performance of CTF modified ANF separators. a) Rate performance of PE, ANF, NCTF/ANF, LCTF/ANF, and SCTF/ANF separators assembled cells at various current densities of 0.5–5 C. b) Long‐term cycling stability of Li//LFP cells at 1 C. c) Charge‐discharge voltage profiles of Li//LFP cells at the 1000^th^ cycle. d) Long‐term cycling stability of Li//LFP cells at 5 C. e) Long‐term cycling stability of Li//LFP cells at 70 °C. f) Cycling performance of Li//NCM811 cells at 0.2 C. g) Cycling performance of graphite//LFP pouch cell using SCTF/ANF separator at 0.2 C. h) Comparison of cycling performances of SCTF/ANF separator assembled cells with that in literature.^[^ ^51^, ^52^, ^53^, ^54^, ^55^, ^56^, ^57^, ^58^, ^59^, ^60^, ^61^, ^62^ ^]^

To evaluate the feasibility of the composite separators applied in practical conditions, the electrochemical performance of Li//LFP cell was investigated. First, the cyclic voltammetry (CV) behaviors of the LMBs equipped with ANF, NCTF/ANF, LCTF/ANF, and SCTF/ANF separators were studied in a voltage window between 2.5 and 4.2 V. As displayed in Figure S34 (Supporting Information), the gap between the oxidation and reduction peaks of LMBs with SCTF/ANF (0.176 V) is smaller than that of ANF, NCTF/ANF, and LCTF/ANF separators. This demonstrates the SCTF/ANF separator's ability to effectively reduce electrochemical polarization while improving the reversibility of Li⁺ redox reactions. The rate performance of cells assembled with different separators were evaluated at current rates ranging from 0.5 C to 5 C (**Figure** **7**a). The SCTF/ANF separators assembled cells exhibit higher average specific capacity than the other separators at current rates of 0.5 C, 1 C, 2 C, 3 C, 4 C, and 5 C. In addition, the SCTF/ANF separator assembled cell has the lowest overpotential at 1 C charge and discharge rate, indicating the weakest polarization and fastest Li^+^ transport (Figure S35, Supporting Information). The long‐term cycling performance of cells with different separators was further investigated. As shown in Figure 7b, the Li//LFP cell assembled with SCTF/ANF separator achieves high capacity retention of 72% after 1000 cycles. Meanwhile, in Figure 7c, the 1000th circle discharge/charge voltage profiles at 1 C exhibit that the SCTF/ANF separator assembled cell has the lowest overpotential of 0.10 V. However, the PE, ANF, NCTF/ANF, and LCTF/ANF separators assembled cells display relatively large overpotentials of 0.76, 0.30, 0.23, and 0.20 V, respectively, and strong polarization due to the rapid irreversible loss of electrolyte and lithium anode. Figure 7d presents the long‐term cycling performance of Li//LFP cells at 5 C. The cell employing the SCTF/ANF separator demonstrates superior cycling stability, delivering an initial capacity of 120.0 mAh g^−1^, and a high CE of 99.9% with capacity retention of 72% after 3000 cycles. As evidenced in Figure 7e, thanks to the excellent thermal stability, the SCTF/ANF‐based cell maintains stable cycling over 200 cycles at 70 °C, retaining a specific capacity exceeding 120.4 mAh g^−1^. Beyond the LFP cathode, the Ni‐rich LiNi_0.8_Co_0.1_Mn_0.1_O_2_ (NCM811) cathode was exploited to further evaluate the performance of SCTF/ANF separator. The assembled Li//NCM811 cell with SCTF/ANF separator exhibits stable capacity retention of 73% at 4.3 V and 0.2 C after 300 cycles, as shown in Figure 7f. Meanwhile, the practical application potential of SCTF/ANF separator is verified through stable cycling performance in pouch cells (initial discharge capacity of 144.2 mAh) employing an artificial graphite anode (17.7 mg cm^−2^) and high‐mass‐loading LFP cathode (32.3 mg cm^−2^) with a low negative/positive (N/P) ratio of 2.4 (Figure 7g). The pouch cell delivers high capacity retention of 83% at 0.2 C after 150 cycles. Compared to recently reported separators^[^ ^51^, ^52^, ^53^, ^54^, ^55^, ^56^, ^57^, ^58^, ^59^, ^60^, ^61^, ^62^ ^]^ and commercial separators (Table S6, Supporting Information), the SCTF/ANF separator exhibits highly competitive overall performance in high‐rate LMBs, high‐voltage LMBs, high‐temperature LMBs and pouch cells (Figure 7h). These results further demonstrate the potential of universal application of SCTF/ANF separators in the field of high‐energy LMBs.

## 3 Conclusion

In conclusion, we successfully designed a ultrasmall hollow CTF nanosphere modified ANF separator to achieve fast, selective and homogeneous transport of Li^+^. By employing CTFs with different geometries for surface coating, combined experimental and computational analyses demonstrate that the mesoscale architecture of the modification layer plays a critical role in regulating ion transport pathways through the separator. The refined ion transport is beneficial for uniform lithium deposition, thus efficiently suppressing the growth of lithium dendrites and boosting the life time of lithium anode. As a consequence, the Li//LFP cell employing the SCTF/ANF separator demonstrates superior cycling stability, delivering an initial capacity of 120.0 mAh g^−1^ at 5 C with 72% capacity retention after 3000 cycles. Moreover, the SCTF/ANF separator assembled pouch cells with high‐mass‐loading LFP cathode (32.3 mg cm^−2^) have remarkable capacity retention of 83% after 150 cycles. Rather than preventing Li dendrites from the delicate molecular‐level design of CTF structures for separator modification, the rational reformation of porous materials provides a new solution to uneven lithium deposition in batteries. This facile strategy can potentially be extended to other modified separators for batteries with better performance.

## Conflict of Interest

The authors declare no conflict of interest.

## Supporting information



Supporting Information

## Data Availability

The data that support the findings of this study are available from the corresponding author upon reasonable request.
