# Supplementary material for: Ultrasmall Hollow Covalent Triazine Framework Nanosphere/Aramid Nanofiber Composite Separator for High‐Energy Lithium Metal Batteries
Source: Adv Sci (Weinh). 2025 Aug 11;12(41):e10757. doi: 10.1002/advs.202510757 (PMC12591181; doi:10.1002/advs.202510757)
Supplement: Supplementary file 1 — Supporting Information [file ADVS-12-e10757-s001.docx]

Supplementary Information

**Ultrasmall Hollow Covalent Triazine Framework Nanosphere/Aramid Nanofiber Composite Separator for High-Energy Lithium Metal Batteries**

Yufei Yang^[a]^, Kaveesha Ihala Kodippili^[b]^, Yun Wang^[a]^, Jintumol Mathew^[b]^, Jie Chen^[a]^, Yi Chen^[a]^, Xingyan Zeng^[a]^, Xuyang Wang^[a]^, Xingping Zhou^[a]^, Petr Král^*[b,c]^, Hui Nie^*[a]^, Xiaolin Xie^[a]^

[a] Y. F. Yang, Y. Wang, J. Chen, Y. Chen, X. Y. Zeng, X. Y. Wang, X. P. Zhou, H. Nie^*^, X. L. Xie

Key Laboratory of Material Chemistry for Energy Conversion and Storage, Ministry of Education, School of Chemistry and Chemical Engineering

Huazhong University of Science and Technology

1037 Luoyu Road, Hongshan District, Wuhan 430074, P.R. China

E-mail: huinie@hust.edu.cn (Dr. H. Nie)

[b] K. I. Kodippili, J. Mathew, P. Král^*^

Department of Chemistry

University of Illinois at Chicago

Chicago, Illinois 60607, United States

E-mail: pkral@uic.edu (Prof. P. Kral)

[c] P. Král^*^

Department of Physics, Pharmaceutical Sciences, and Chemical Engineering

University of Illinois at Chicago

Chicago, Illinois 60607, United States

E-mail: pkral@uic.edu (Prof. P. Kral)

**Contents**

1. Supplemental Experimental3
2. Supplemental Figures11
3. Supplemental Tables35
4. Supplemental References39

**Supplemental Experimental**

**Materials**

All the materials and solvents were purchased from commercial sources without further purification. 1-methyl-2-pyrrolidinone (NMP), dimethyl sulfoxide (DMSO), N, N-dimethylformamide (DMF), potassium tert-butoxide (t-BuOK), methanol (MeOH) and tetraethoxysilane (TEOS) were purchased from Sinopharm Chemical Reagent Co. (Shanghai, China). 3-aminopropyltriethoxysilane (APTES) and hydrofluoric acid (HF) were obtained from Aldrich Chemical Co. (Shanghai, China). Polyvinylidene fluoride (PVDF, *M*_w_ ~1 000 000), LiFePO_4_, and Super-P were purchased from Canrd New Energy Technology Co. Ltd. (Guangdong, China). The liquid electrolyte (1 mol L^-1^ LiPF_6_) in a mixed solvent of ethylene carbonate (EC) and diethyl carbonate (DEC) with a volume ratio of 1:1 was purchased from Duoduo Chemical Technology Co. Ltd. (Suzhou, China). The liquid electrolyte (1 mol L^-1^ LiPF_6_) in a mixed solvent of EC and diethyl DEC (volume ratio of 1:1) with fluoroethylene carbonate was purchased from Duoduo Chemical Technology Co. Ltd. (Suzhou, China). The liquid electrolyte (0.5 M LiBOB) in propylene carbonate was purchased from Duoduo Chemical Technology Co. Ltd. (Suzhou, China). The polyethylene (PE) separator was obtained from Senior Technology Materials Co. Ltd. (Shenzhen, China).

**Synthesis of SiO_2_ nanospheres**

SiO_2_ nanospheres were synthesized according to the reported Stöber method^[1]^. In brief, for the SiO_2_ with a size of about 180 nm, deionized water (86.4 g), ethanol (790.0 g) and ammonia solution (28.2 g) were mixed with stirring. TEOS (62.48 g) was added dropwise to the above mixture with stirring at 60 ℃ for 8 h. After the reaction, the SiO_2_ nanospheres were separated by centrifugation and washed several times with ethanol by centrifugation and redispersion. Then, the purified SiO_2_ nanospheres were obtained after drying under vacuum for 24 h. For the SiO_2_ nanospheres with a size of about 30 nm, deionized water (81.4 g), ethanol (815.0 g) and ammonia solution (12.2 g) were mixed with stirring. TEOS (62.48 g) was added dropwise to the above mixture with stirring at 60 ℃ for 8 h. After washing several times with ethanol by centrifugation and redispersion, it was dried under vacuum for 24 h.

**Synthesis of amino-modified SiO_2_ nanospheres (NH_2_-SiO_2_)**

According to the method reported by Wang and co-workers^[2]^, the SiO_2_ nanospheres with a size of about 180 nm (5.0 g) were dispersed in ethanol (900 mL) by sonication. Then, APTES (1.5 mL) and ethanol (100 mL) were mixed, and the mixture was added slowly to SiO_2_ nanospheres ethanol dispersion under vigorous stirring at 32 ℃. The mixture was allowed to react for 12 h to obtain the amino group-modified SiO_2_ nanospheres. For the SiO_2_ nanospheres with a size of about 30 nm, APTES (3 mL) in ethanol (100 mL) was added to SiO_2_ nanospheres (5 g) ethanol dispersion (900 mL). The obtained products were separated by centrifugation. After washing with deionized water and ethanol by centrifugation and redispersion, the modified SiO_2_ nanospheres were dried under vacuum for 24 h.

**Synthesis of core-shell structure NH_2_-SiO_2_@CTF**

Large-sized core-shell structure NH_2_-SiO_2_@CTF (LCTF-CS) and ultrasmall-sized core-shell structure NH_2_-SiO_2_@CTF (SCTF-CS) were synthesized according to modified protocols reported by Tan et al^[3]^. For the LCTF-CS nanospheres, firstly, a beaker was charged with terephthalaldehyde (0.067 g) and DMSO (30 mL), and the prepared aldehyde solution was marked as solution A. Secondly, 180 nm NH_2_-SiO_2_ (0.5 g) was ultrasonically dispersed in DMSO (70 mL) at room temperature. Then, solution A (7.14 mL) was added dropwise to the above solution by a peristaltic pump with feed rate of 0.05 mL min^-1^ and reacted at 60 ℃ for 12 h. After that, terephthalamidine dihydrochloride (0.056 g) and cesium carbonate (0.24 g) were added to the reaction system. After reaction at 60 ℃ and 80 ℃ for 12 h, respectively, core-shell structure with prepolymer grown on NH_2_-SiO_2_ nanospheres was obtained. After the addition of terephthalamidine dihydrochloride (0.179 g) and cesium carbonate (0.480 g), solution A (12.86 mL) was added dropwise by a peristaltic pump with feed rate of 0.05 mL min^-1^, and reacted at 100 ℃ and 120 ℃ for 24 h, respectively. The obtained products were washed for three times with deionized water and DMF by centrifugation. The purified LCTF-CS samples were freeze-dried for 24 h. For the SCTF-CS nanospheres, terephthalaldehyde (0.1005 g) and DMSO (30 mL) were mixed as solution A. Then, solution A (7.14 mL) was added dropwise to the DMSO (70 mL) including 30 nm NH_2_-SiO_2_ (0.5 g) by a peristaltic pump with feed rate of 0.05 mL min^-1^ and reacted at 60 ℃ for 12 h. After that, terephthalamidine dihydrochloride (0.084 g) and cesium carbonate (0.036 g) were added to the reaction system. After reaction at 60℃ and 80℃ for 12 h, terephthalamidine dihydrochloride (0.2685 g) and cesium carbonate (0.720 g), solution A (12.86 mL) were added dropwise by a peristaltic pump with feed rate of 0.05 mL min^-1^, and reacted at 100 ℃ and 120 ℃ for 24 h, respectively. After washing with deionized water by centrifugation and redispersion, the SCTF-CS samples were dried under vacuum for 24 h.

**Synthesis of LCTF and SCTF**

1 g LCTF-CS or SCTF-CS samples were dispersed in 10 g HF (49 wt%) and the suspensions were kept stirring at room temperature for 24 h. After the reaction, the suspensions were suction-filtrated and washed with deionized water for 5 h. The obtained samples were freeze-dried for 24 h, giving large-sized CTF nanosphere (LCTF) or ultrasmall-sized CTF nanosphere (SCTF).

**Synthesis of NCTF**

CTF sheet-like structure (NCTF) was synthesized according to modified protocols reported by Tan et al^[4]^. 1,4-phthalaldehyde (0.0672g), terephthalamidine dihydrochloride (0.2352g), and Cs_2_CO_3_ (0.7168g) were dissolved in a mixture of 7.5 mL of DMSO. The mixture was stirred at 60 °C, 80 °C, 100 °C and 120 °C under air atmosphere for 12 hours. After the reaction, the solution was filtered, washed with DMF and deionized water, then dried to obtain yellow NCTF powder.

**Preparation of CTF/ANF composite separator**

First, 0.015g CTF and 0.005g PVDF (weight ratio of CTF: PVDF = 3:1) were dispersed in NMP (8.3 g) with ultrasound and stirring for 6 h. The obtained slurry was blade-coated on a glass substrate with the liquid film thickness of 20 μm and then dried at 100 ℃ for 20 min under vacuum. Second, *t*-BuOK (1.5 g) was dispersed in MeOH (1.5 g) and DMSO (45.5 g) with ultrasound, and then poly (p-phenylene terephthamide) (1.5 g) was added to the above solution. After reaction at 70 ℃ for 1 h with vigorous stirring, an aramid nanofiber (ANF) solution was obtained^[5]^. The ANF solution was then cast onto the glass substrate with CTF by blade coating with a liquid film thickness of 200 μm. After standing for 30 s, the substrate with CTF and ANF was immersed in water for phase inversion. The obtained CTF/ANF separators were transferred into ethanol and then dried at 100 ℃.

**Preparation of LFP cathode**

PVDF **(**0.16 g), Super-P (0.16 g) and LiFePO_4_ (LFP, 1.28 g) were mixed and ground for 30 min, then the mixed powders were dispersed in NMP (10 mL) with vigorous stirring for 5 h. The obtained slurry was blade-coated on Al foil and dried at 60 ℃. Finally, the cathode-coated Al foil was cut into discs with a diameter of 12 mm.

**Characterization**

The structure of NCTF, LCTF and SCTF was determined by powder X-ray diffraction (PXRD, SmartLab-SE) with Cu-Kα radiation. FI-IR spectra were obtained by using fourier-transformed infrared spectrometer (VERTEX 70). The thickness of NCTF was obtained by atomic force microscope (AFM, Jupiter XR). The morphology of NCTF, LCTF and SCTF was characterized by field emission transmission electron microscopy (FE-TEM, HT7700, Hitachi). The thermal properties of NCTF/ANF, LCTF/ANF and SCTF/ANF separators were evaluated using the thermal gravimetric analysis (TGA) instrument (4000, Perkin-Elmer) at the rate of 10 °C/min under a nitrogen atmosphere up to 800 °C. The glass transition temperature was evaluated using differential scanning calorimetry (DSC) instrument (Q2000, TA). The Brunauer-Emmett-Teller (BET) specific surface area and pore size distribution of CTFs and CTF-modified ANF separators were measured on ASAP2420-4MP at 77 K. Surface morphology of different separators were analyzed by field emission scanning electron microscopy (Nova Nano FE-SEM 450). The cross-sectional structure of the separator was examined by focused ion beam scanning electron microscopes (FIB-SEM, Thermo Fisher Scientific, Helios 5). Time-of-flight secondary ion mass spectrometry (TOF-SIMS, Thermo Fisher Scientific, Helios 5) was utilized to quantify the rate and uniformity of ion transport. The contact angles of the separators toward liquid electrolytes were measured using a contact angle tester (OCA20). Raman spectra (background corrected) were tested on Raman spectrometer (ATR8100) with a laser of 785 nm. Tensile experiments were carried out on a SANS CMT-4104 universal testing machine with 20 mm/min crosshead speed at ambient conditions, and the sample size was about 10×0.7 cm. Chemical composition of Li electrode and different separators were analyzed by X-ray photoelectron spectroscopy (XPS, AXIS SUPRA+). The electrochemical mechanisms were determined by in-situ attenuated total reflection-Fourier transform infrared (ATR-FTIR, Bruker) spectrometer instrument. In-situ ATR-FTIR cell was purchased from the Beijing Scistar Technology Co. Ltd.

The electrolyte uptake (EU) was evaluated by measuring the weight of separators before and after immersing into the liquid electrolyte. The electrolyte retention (ER) was evaluated by measuring the weight of separators after it has absorbed the electrolyte and rested for a period of time.

$$\begin{aligned} EU\left( \% \right)=\frac{W_{2}-W_{1}}{W_{1}}\times100\%\#\left( 1 \right) \end{aligned}$$

$$\begin{aligned} ER\left( \% \right)=\frac{W_{3}-W_{1}}{W_{2}-W_{1}}\times100\%\#\left( 2 \right) \end{aligned}$$

where $W_{1}$ and $W_{2}$ are the weight of the separators before and after being immersed in the liquid electrolyte. And $W_{3}$ is the weight of the separator after it had absorbed the electrolyte and rested for a period of time.

The porosity was obtained by measuring the weight of separators before and after immersion in *n*-butanol:

$$\begin{aligned} Porosity=\frac{W_{5}-W_{4}}{V\times\rho}\times100\%\#\left( 3 \right) \end{aligned}$$

where $W_{4}$ and $W_{5}$ are the weight of separators before and after immersion in *n*-butanol, *V* is the volume of separators, and *ρ* is the density of *n*-butanol (*ρ* =0.81 g cm^-3^).

**Electrochemical measurements**

CR2032-type coin cells were used to test the electrochemical performance with different separators. Pouch cells with LFP cathode and graphite anode were prepared using a 5 cm × 8 cm pouch cell.

**Electrochemical stabilization window**

Linear sweep voltammetry (LSV) was carried out on an electrolyte-soaked separator sandwiched between lithium metal and stainless steel with a scan rate of 10 mV s^-1^ on an AUTOLAB (PGSTAT302N).

**Ionic conductivity (σ)**

The electrochemical impedance spectroscopy (EIS) was used to calculate the ionic conductivity by the following equation 4. The bulk resistance was determined as the extrapolated high frequency intercept with the real x-axis of the Nyquist plot. The sloping line represents the diffusion process of Li^+^, which is described by the Warburg impedance.

$$\begin{aligned} \sigma= \frac{d}{R_{d}\times S}\#\left( 4 \right) \end{aligned}$$

where $d$ and $S$ are the thickness and area of the separators, and $R_{d}$ is the bulk ohmic resistance of the electrolyte.

**Lithium ion transference number (**$\boldsymbol{t}_{\boldsymbol{Li}^{\mathbf{+}}}$**)**

The $t_{{Li}^{+}}$ was evaluated by combining chronoamperometry and EIS analysis using lithium symmetric cells. $t_{{Li}^{+}}$ was calculated according to the following equation 5:

$$\begin{aligned} t_{{Li}^{+}}=\frac{I_{s}\left( \Delta V-I_{0}R_{el}^{0} \right)}{I_{0}\left( \Delta V-I_{s}R_{el}^{s} \right)}\#\left( 5 \right) \end{aligned}$$

where $I_{0}$ and $I_{s}$ are the initial current and steady-state current, $R_{el}^{0}$ and $R_{el}^{s}$ are the interfacial resistance before and after polarization, respectively, and potential ($\Delta V$) is 10 mV.

**Cyclic voltammetry (CV)**

The CV profiles of LFP||Li cells were tested between 4.2 and 2 V with a scan rate of 0.1 mV s^-1.^

**Determination of the electrical tortuosity**

According to the method reported by Sun et al^[6]^, tortuosity was obtained through the EIS test.

$$\begin{aligned} R_{series}=R_{component}+R_{interfacial}\#\left( 6 \right) \end{aligned}$$

$$\begin{aligned} R_{cell}=R_{series}+\frac{\tau_{e}^{2}d}{\sigma_{0}PS}\#\left( 7 \right) \end{aligned}$$

where $R_{component}$ is electrical resistance of the coin cell’s component, $R_{interfacial}$ is an interfacial resistance between electrode and electrolyte, $R_{cell}$ is the coin cell resistance interpreted from impedance test, $\tau_{e}$ is electrical tortuosity, *d* (in m) is the thickness of the separator in the coin cell, $\sigma_{0}$ is the conductivity of the bulk electrolyte, $P$ is porosity and $S$ (in m^2^) is the electrode’s area. $R_{series}$ is the resistance of the series circuit representing the designed coin cell with zero thickness separator. It includes the resistance of the coin cell components (empty cell) (spring, two spacers, positive and negative cases) and the interfacial resistance between spacers and electrolytes when the resistance of the electrolytes is zero. $R_{series}$ can be determined from the designed coin cells by gradually reducing the separator layer number and extending the measured data to zero separator layer. Furthermore, $R_{component}$ can be obtained by measuring the resistance of the empty cell. Therefore, the electrical tortuosity of the separator can be derived from equation 8.

$$\begin{aligned} \frac{{dR}_{cell}}{{dL}_{S}}=\frac{\tau_{e}^{2}d}{\sigma_{0}PS}\#\left( 8 \right) \end{aligned}$$

The ionic resistance of the separator filled with electrolytes was determined by EIS testing. By measuring the EIS of single-layer and stack-layered separators, the slope of the fitted curve of the $R_{cell}$ with increased thickness of the separator was obtained (**Figure S10**). Then the tortuosity of different separators was calculated by equation 8.

**Molecular dynamics simulations**

Classical molecular dynamics simulations were carried out to model the experimental systems. The diethyl carbonate and Li^+^ were parametrized by the CHARMM36 force field,^[7]^ while ethylene carbonate and PF_6_^-^ were parametrized with CGenFF^[8,9]^ and MP2 calculations, respectively. The atomic charges of CTF layer were obtained using the ESP method at the MP2/6-31G* level in diethyl carbonate and ethylene carbonate implicit solvents. The systems were simulated in an NPT ensemble (T = 300 K, p = 1 bar) using a Langevin dynamics (γ_Lang_ = 1 ps^−1^) in NAMD3.^[10]^ The particle-mesh Ewald (PME)^[11]^ method was used to evaluate the long-range Coulombic interaction. Time step was set to 2 fs. After 100,000 steps of minimization, the systems were simulated in an electric field of $E = 2.17 mV/nm$. Currents and current density measurements were obtained by averaging over the final trajectories after the systems reached a steady state (pore A, through the layers160 ns and pore B, along the layers 280 ns). The trajectories and snapshots were visualized by VMD.^[12]^

**Density functional theory (DFT) calculations**

The binding energy was calculated using density functional theory (DFT), defined as the total energy of the complex minus the sum of the total energies of its constituent monomers.

$$\begin{aligned} E_{\text{binding}}=E_{\text{complex}}-\left( E_{\text{monomerA}}+E_{\text{monomerB}} \right)\#(9) \end{aligned}$$

Geometry optimizations and vibrational frequency analyses were conducted for both the complex and the monomers to identify the minimum energy configurations, with zero-point energy corrections applied to improve the accuracy of the calculated energies.

**Supplementary Figures**


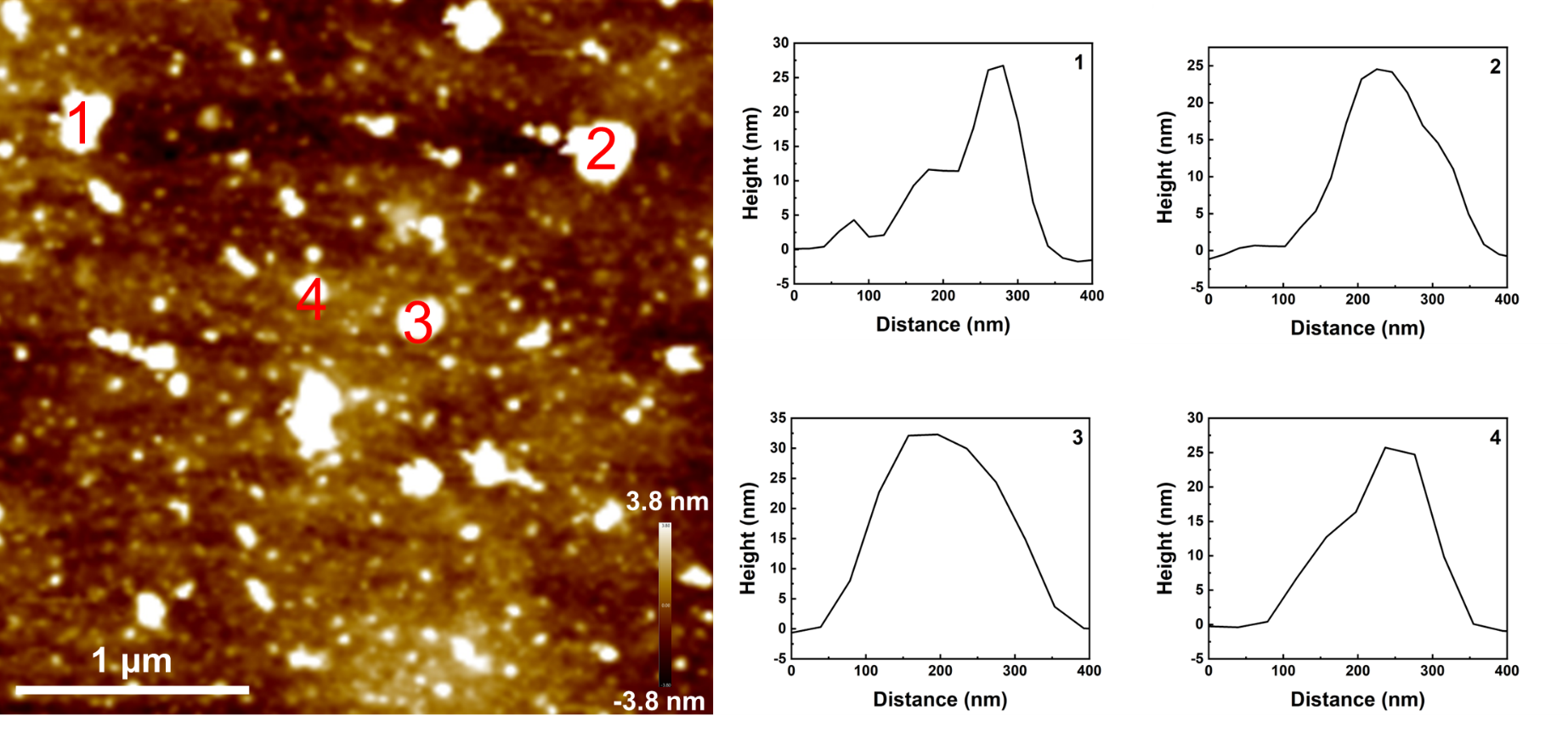


**Figure S1.** AFM topography and the corresponding height profiles of the NCTF.

**
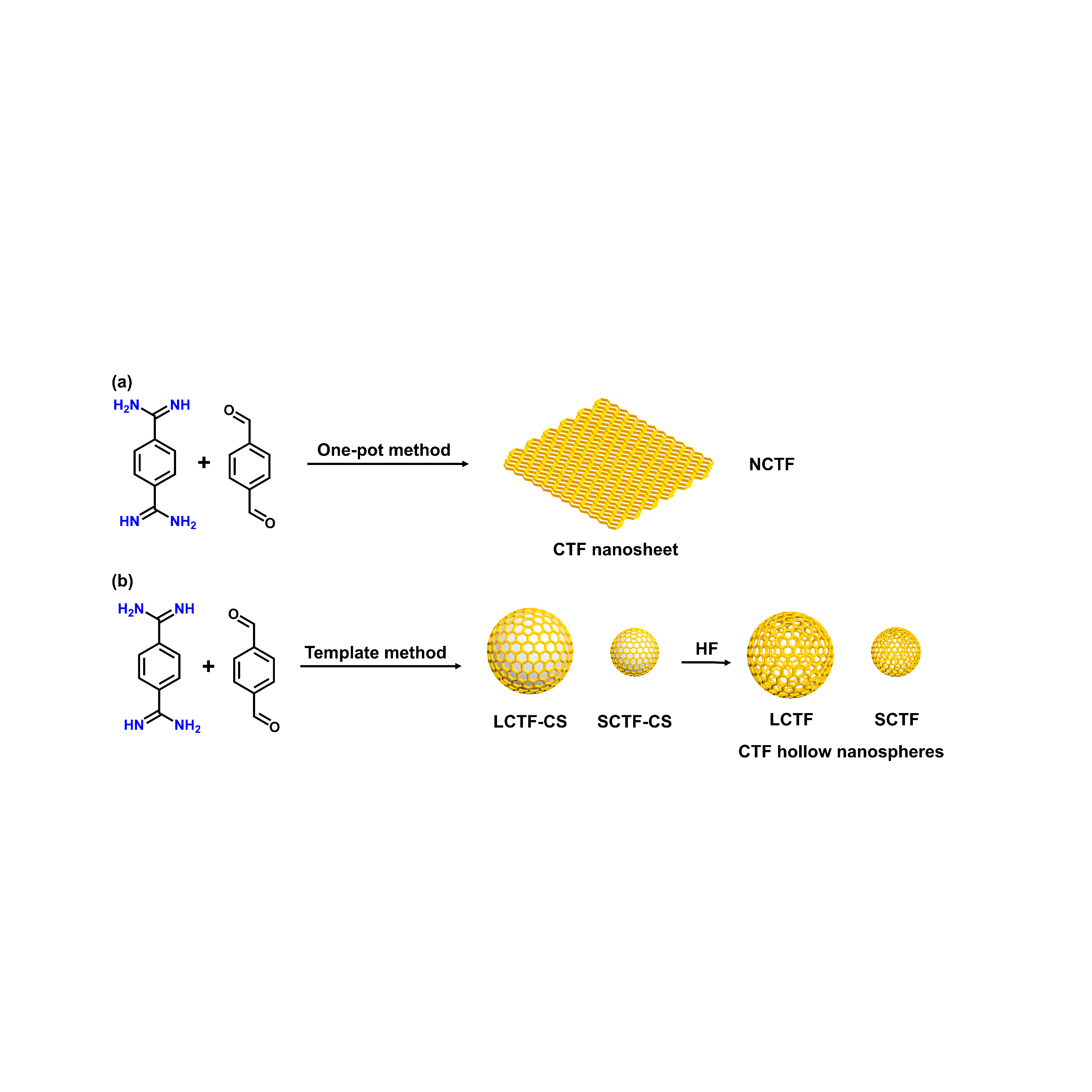
**

**Figure S2.** Schematic synthesis routes of (**a**) NCTF, (**b**) LCTF and SCTF.





**Figure S3.** TEM images of (**a**) LCTF-CS and (**b**) SCTF-CS.

**
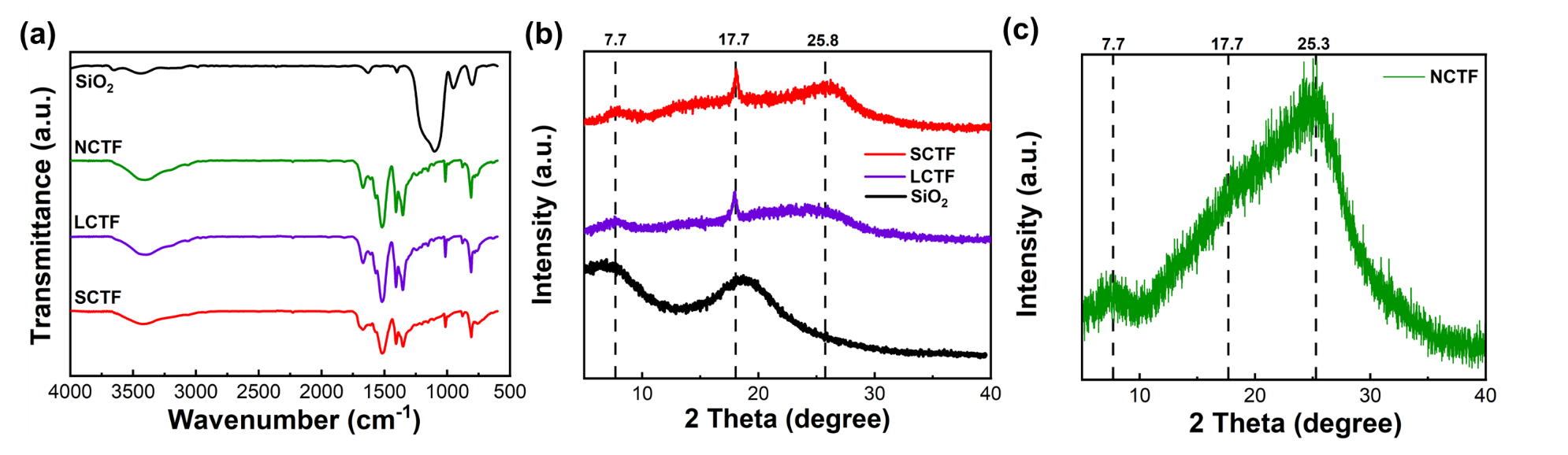
**

**Figure S4.** (a) FT-IR spectra of NCTF, LCTF, SCTF and SiO_2_. (b, c) PXRD patterns, of NCTF, LCTF, SCTF and SiO_2_.


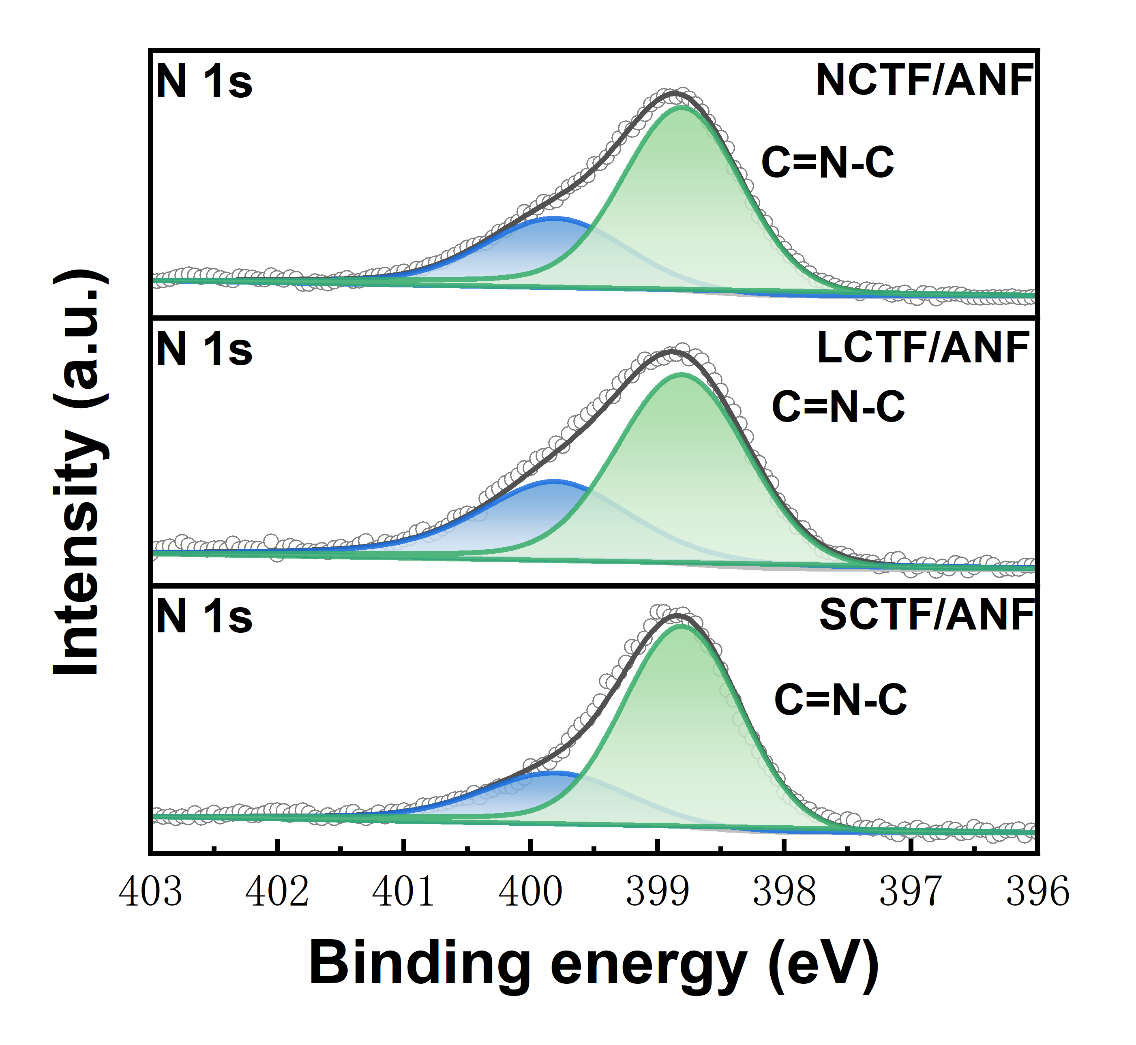


**Figure S5** XPS spectra of NCTF/ANF, LCTF/ANF and SCTF/ANF separators.

**
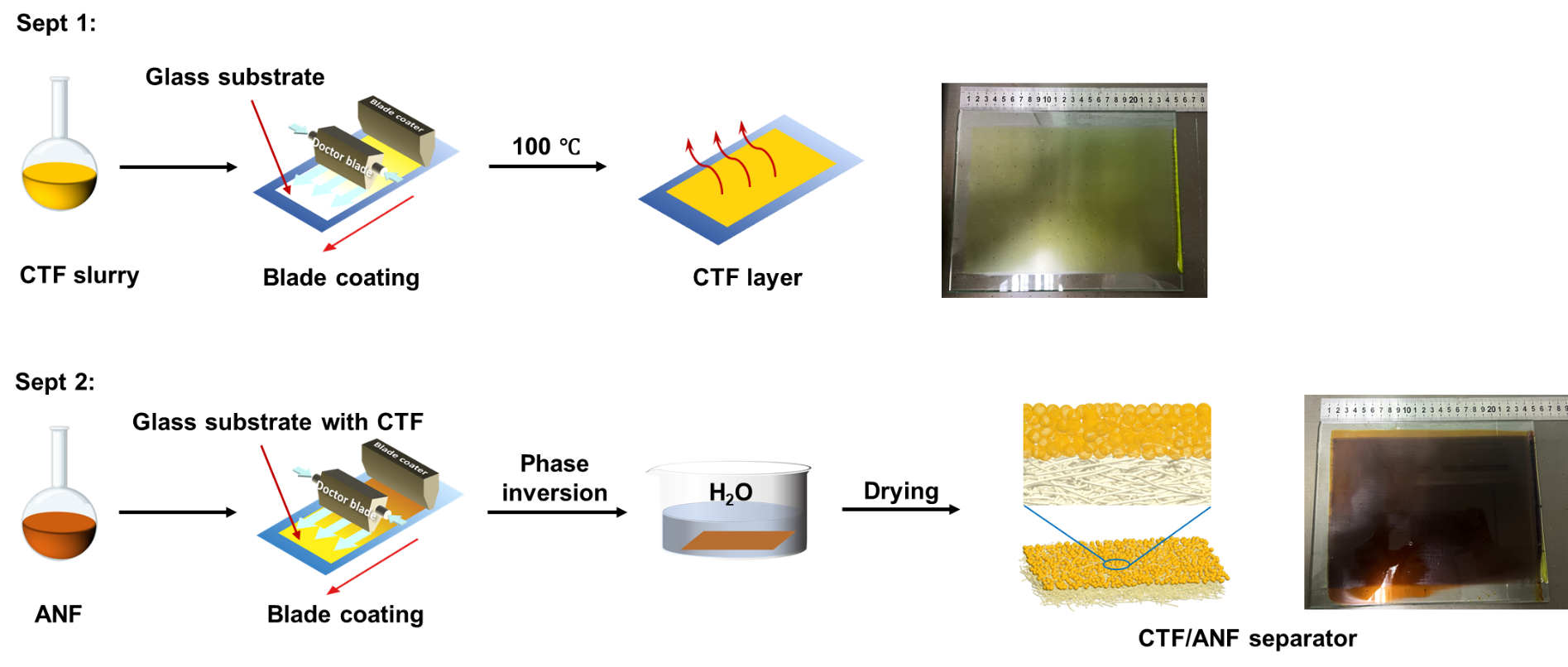
Figure S6.** Fabrication of CTF/ANF separators using the two-step film-casting process combined with a phase inversion approach. (Experimetal details are described in supplemental experimental)


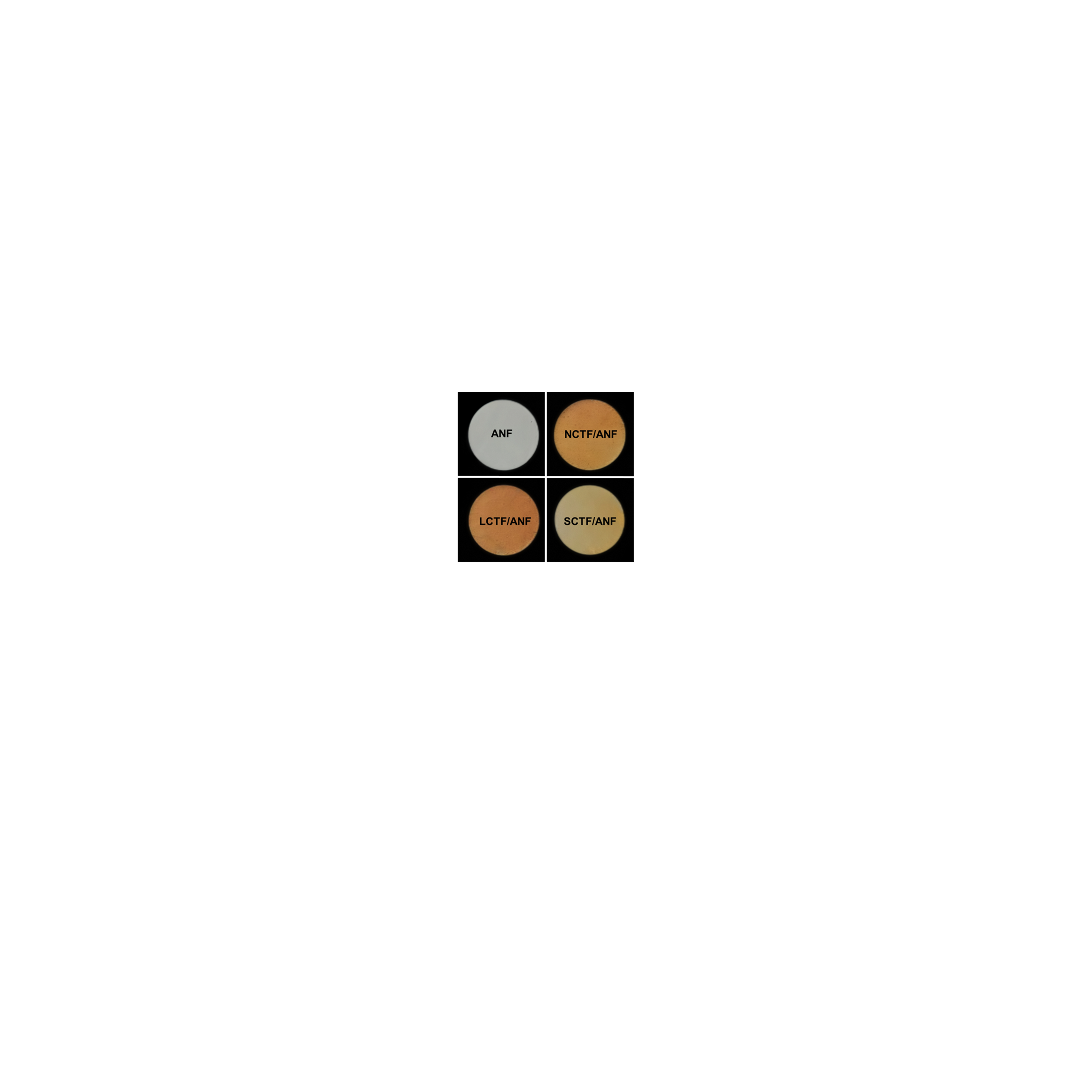


**Figure S7.** Photographs of ANF, NCTF/ANF, LCTF/ANF and SCTF/ANF separators.

**

**

**Figure S8.** Top-view FE-SEM images of the (**a**) NCTF/ANF and (**b**) LCTF/ANF separators.


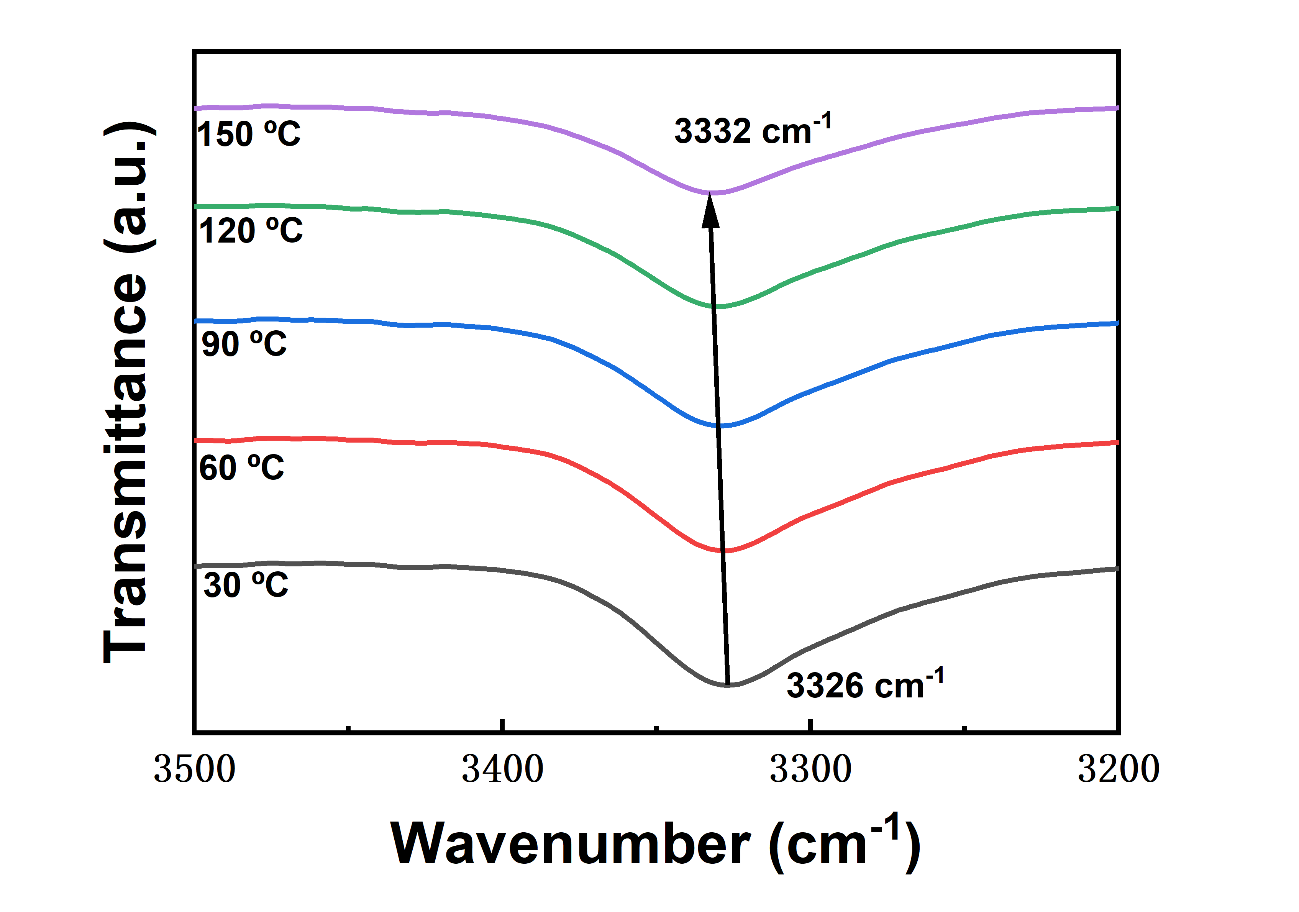


**Figure S9.** Temperature-dependence of FT-IR spectra of CTF/ANF separators.


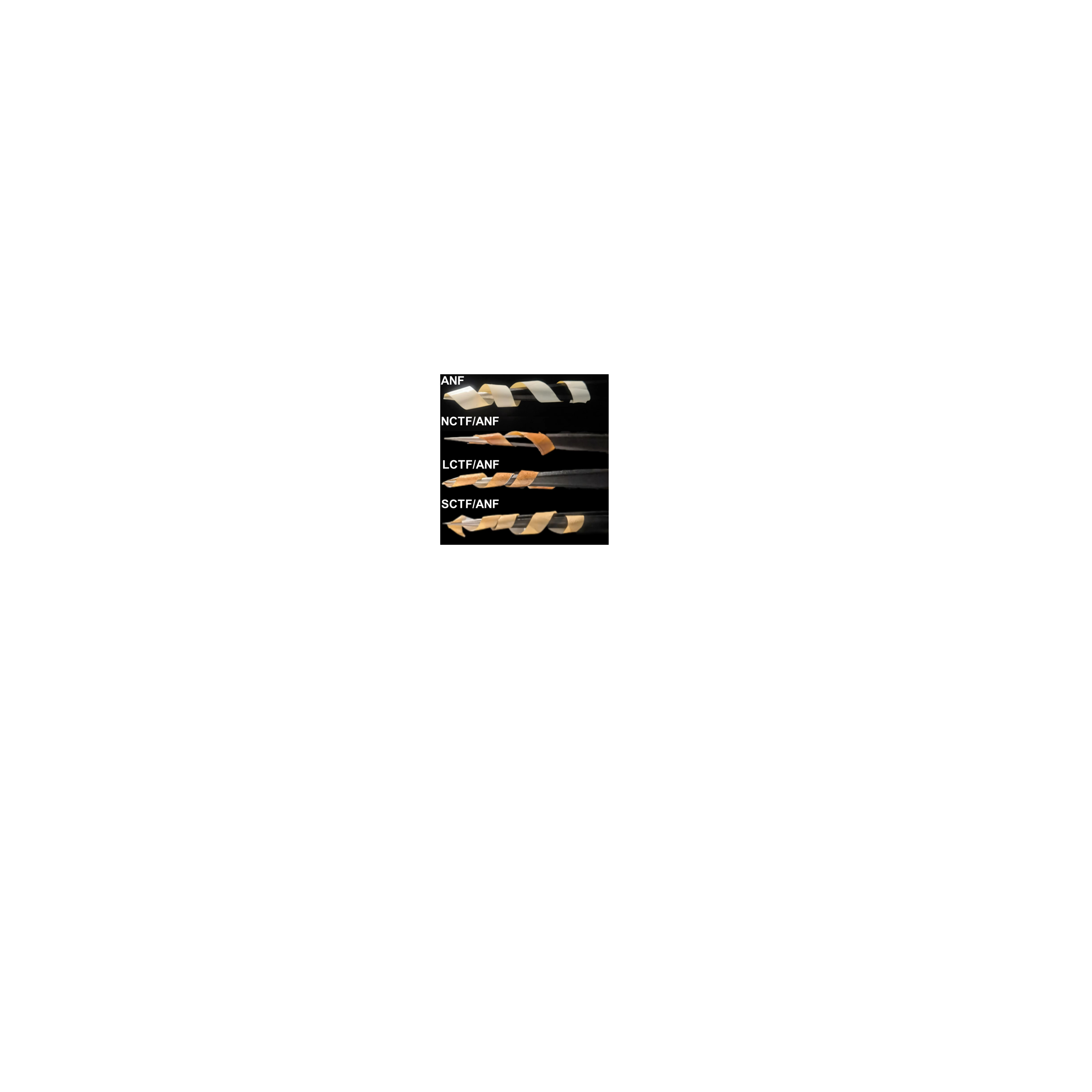


**Figure S10.** Photographs of ANF, NCTF/ANF, LCTF/ANF and SCTF/ANF separators under folding conditions.


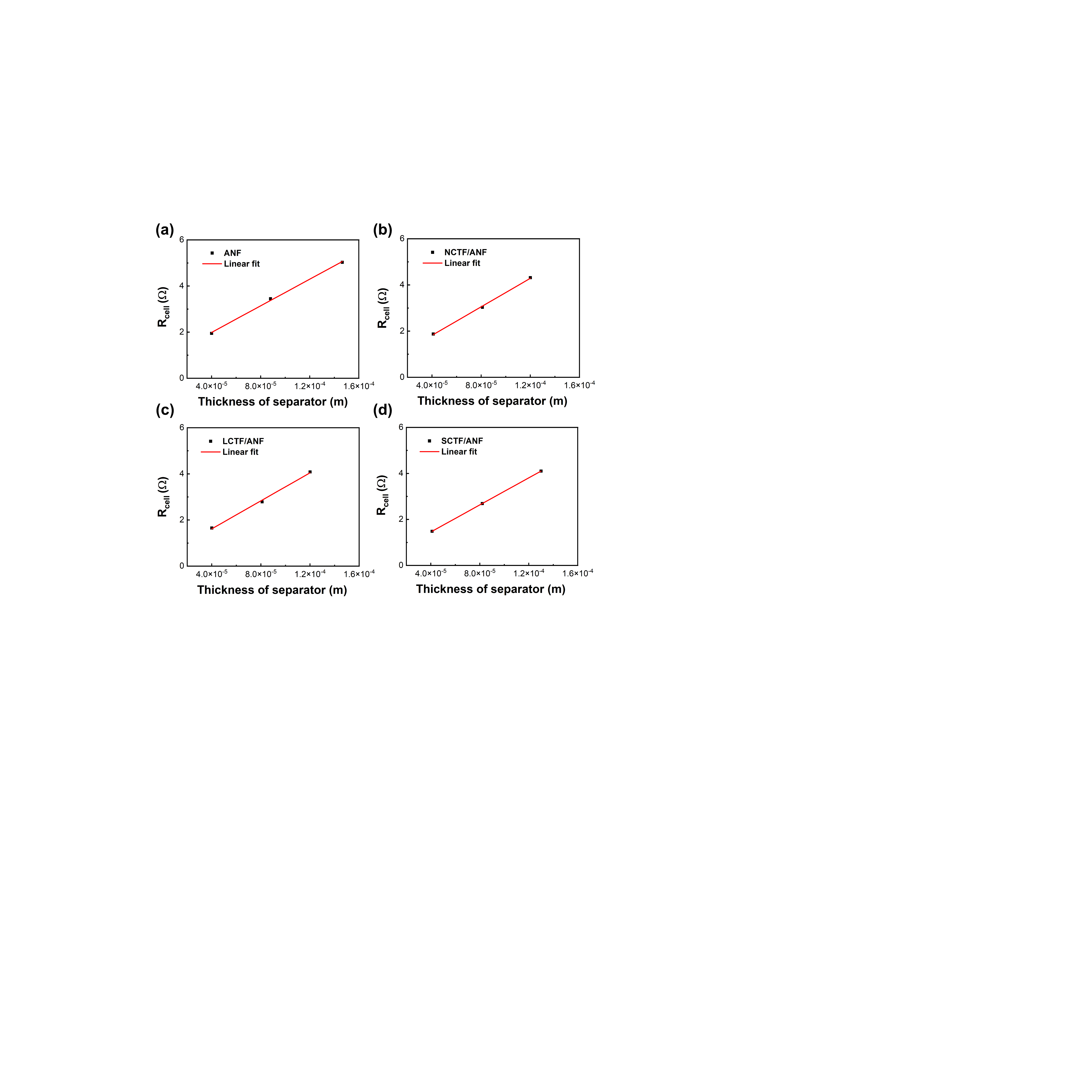


**Figure S11.** The fitted curve of $R_{cell}$ with increased thickness of the (**a**) ANF, (**b**) LCTF/ANF, (**c**) NCTF/ANF and (**d**) SCTF/ANF separators.


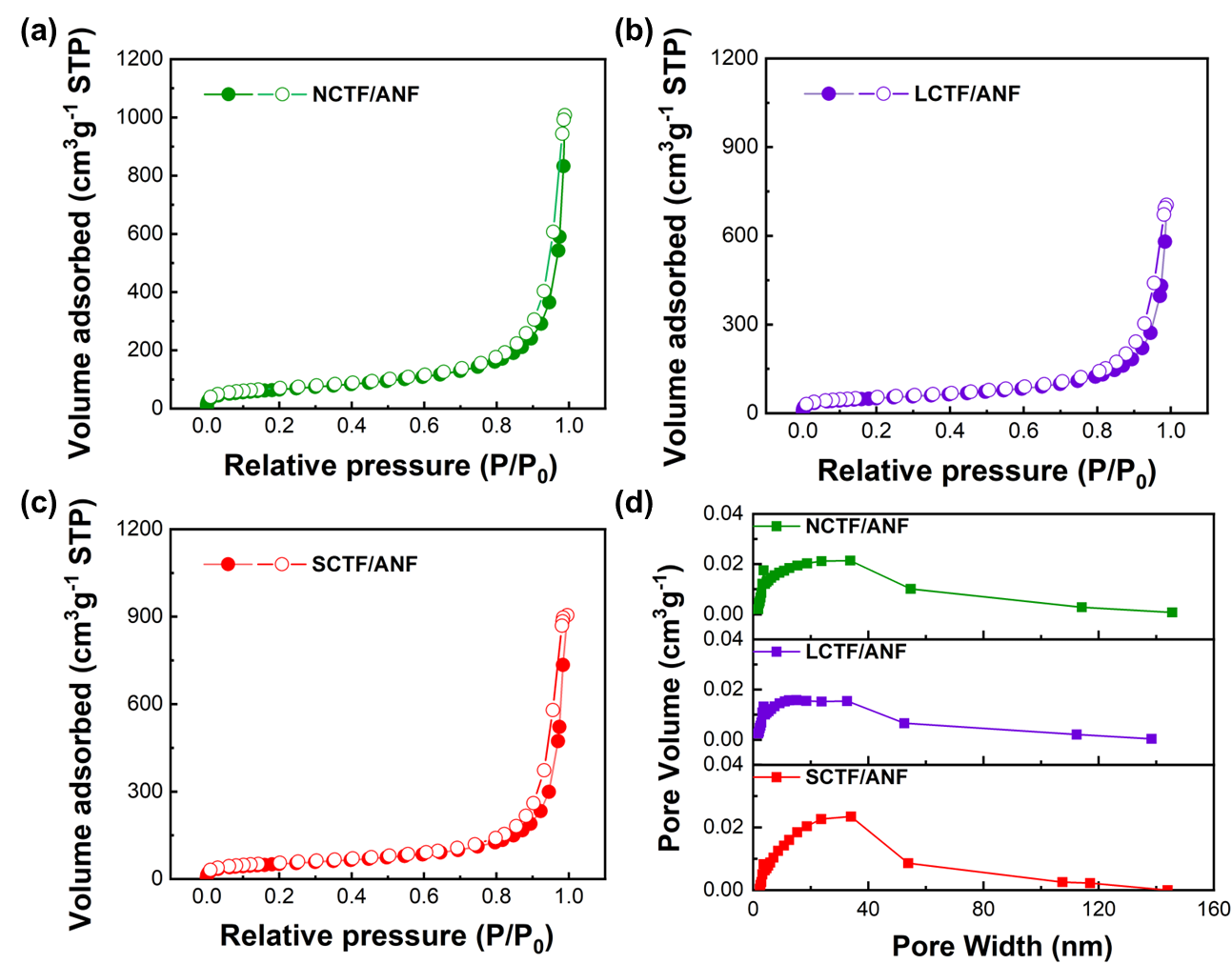


**Figure S12.** N_2_ adsorption and desorption isotherms of (**a**) NCTF/ANF, (**b**) LCTF/ANF and (**c**) SCTF/ANF separators. (**d**) Pore size distributions of NCTF/ANF, LCTF/ANF and SCTF/ANF separators.


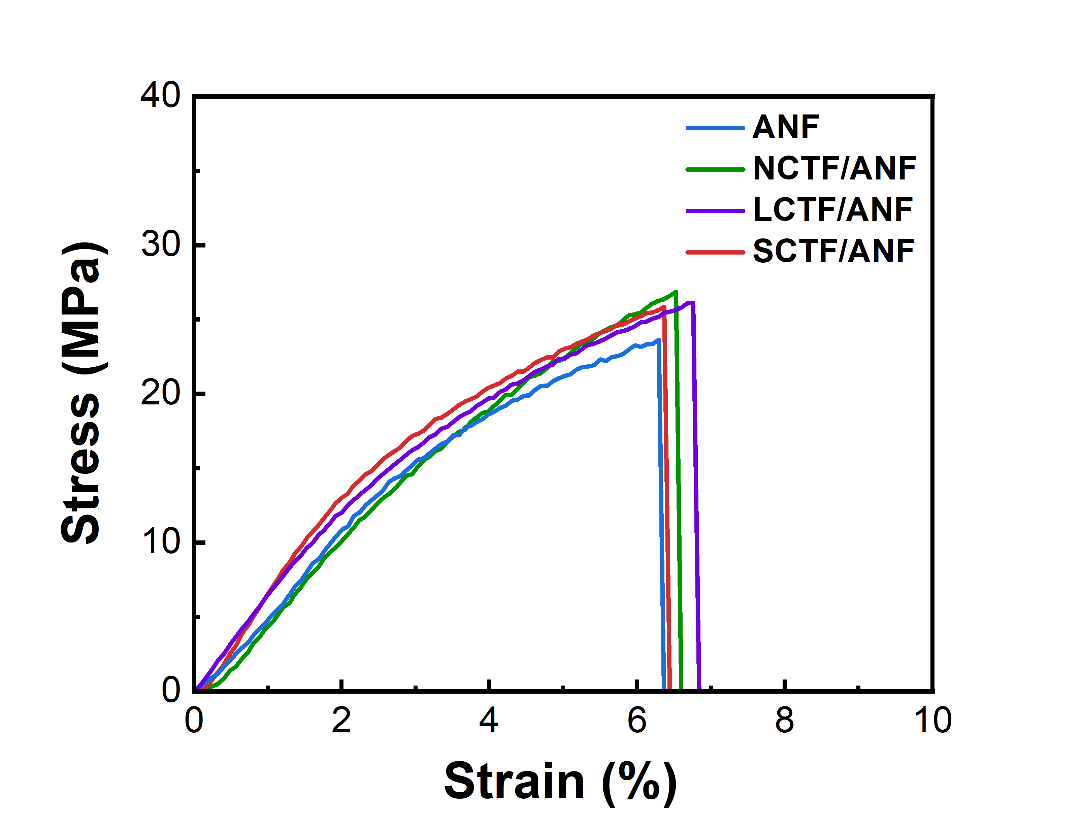


**Figure S13** The tensile-strain curves of ANF, NCTF/ANF, LCTF/ANF and SCTF/ANF separators.

**
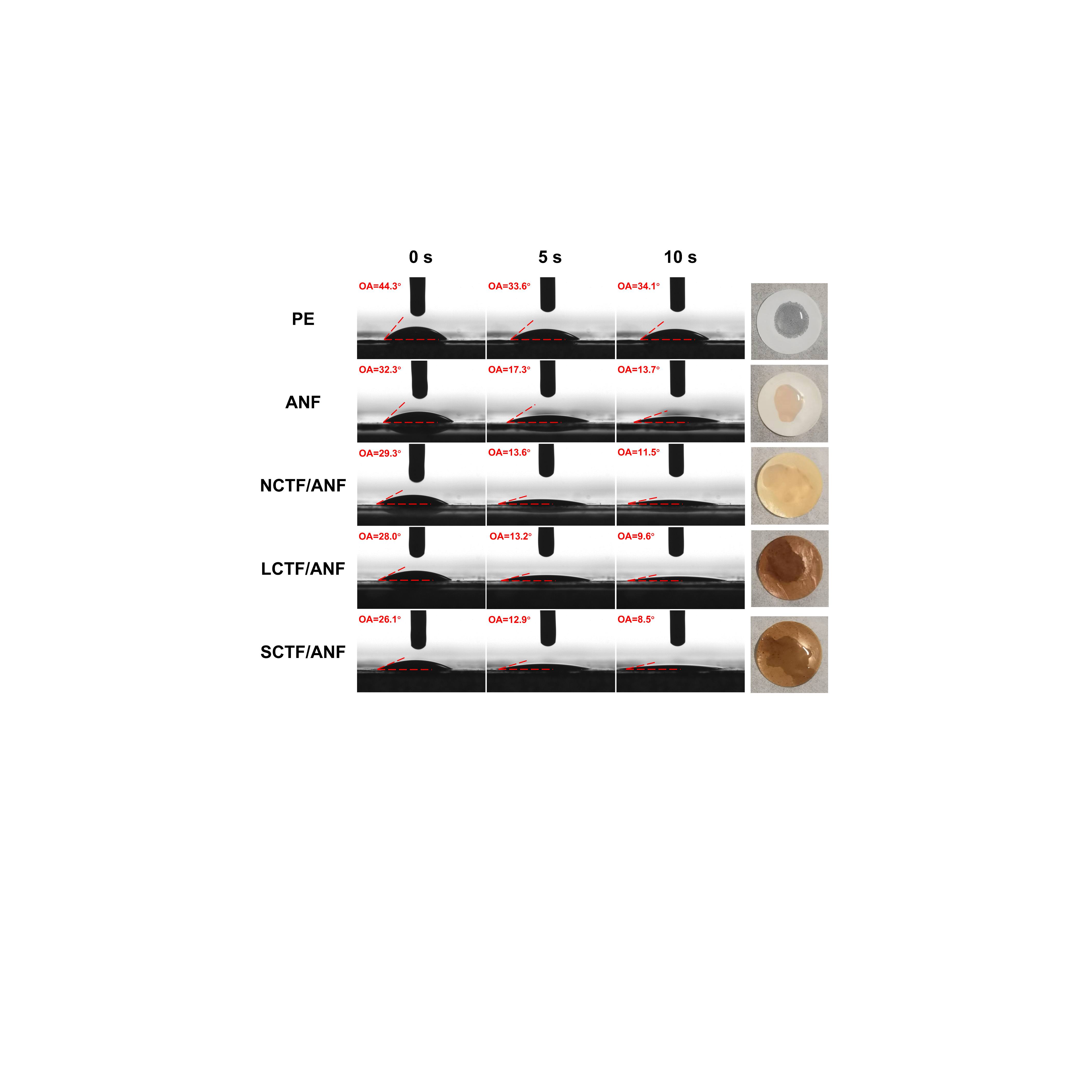
**

**Figure S14.** The electrolyte contact angles of PE separator, the CTF coating side of ANF, NCTF/ANF, LCTF/ANF and SCTF/ANF separators.


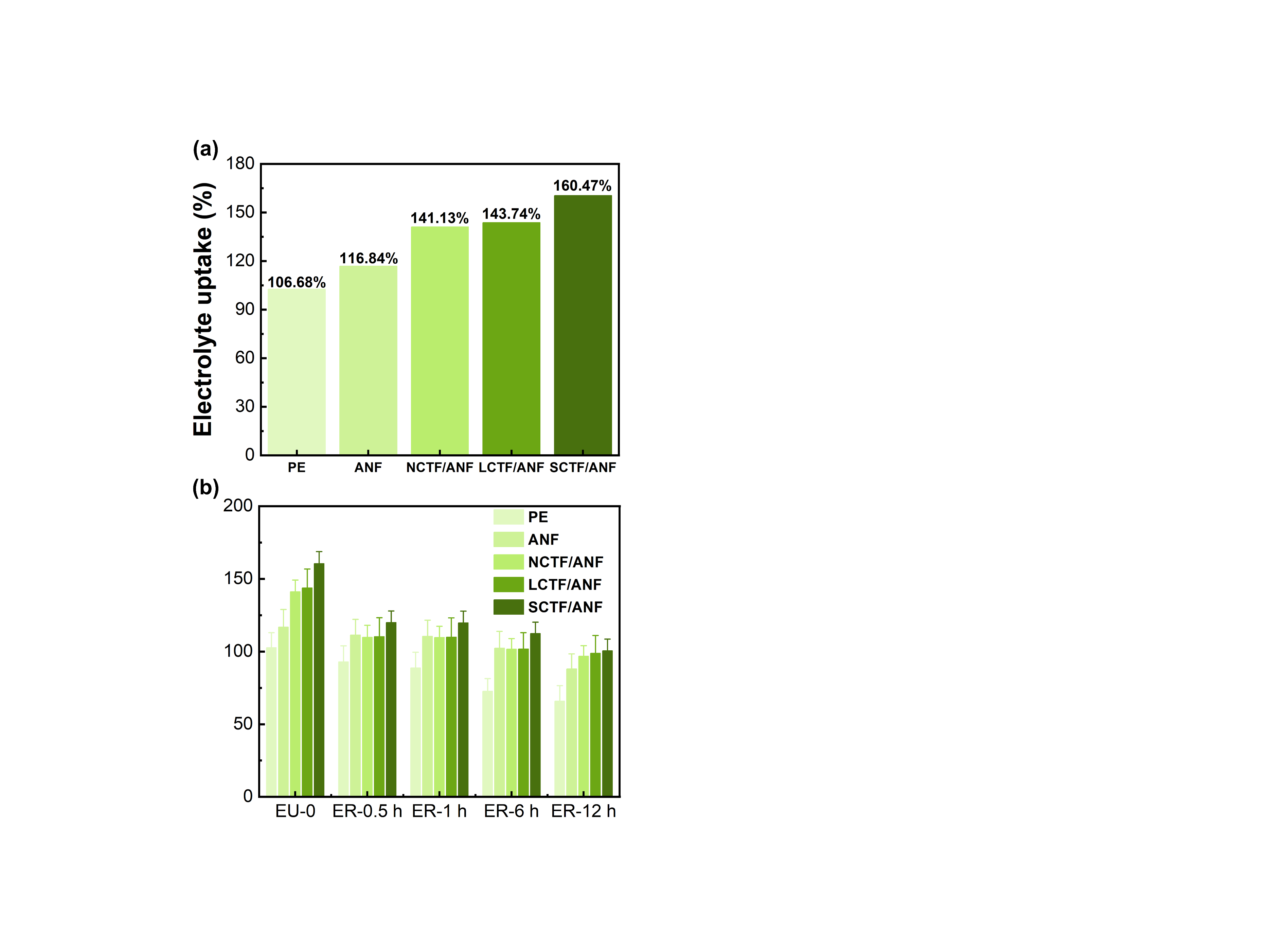


**Figure S15.** (**a**) Electrolyte uptake and (**b**) Electrolyte retention of the PE, ANF, NCTF/ANF, LCTF/ANF and SCTF/ANF separators.


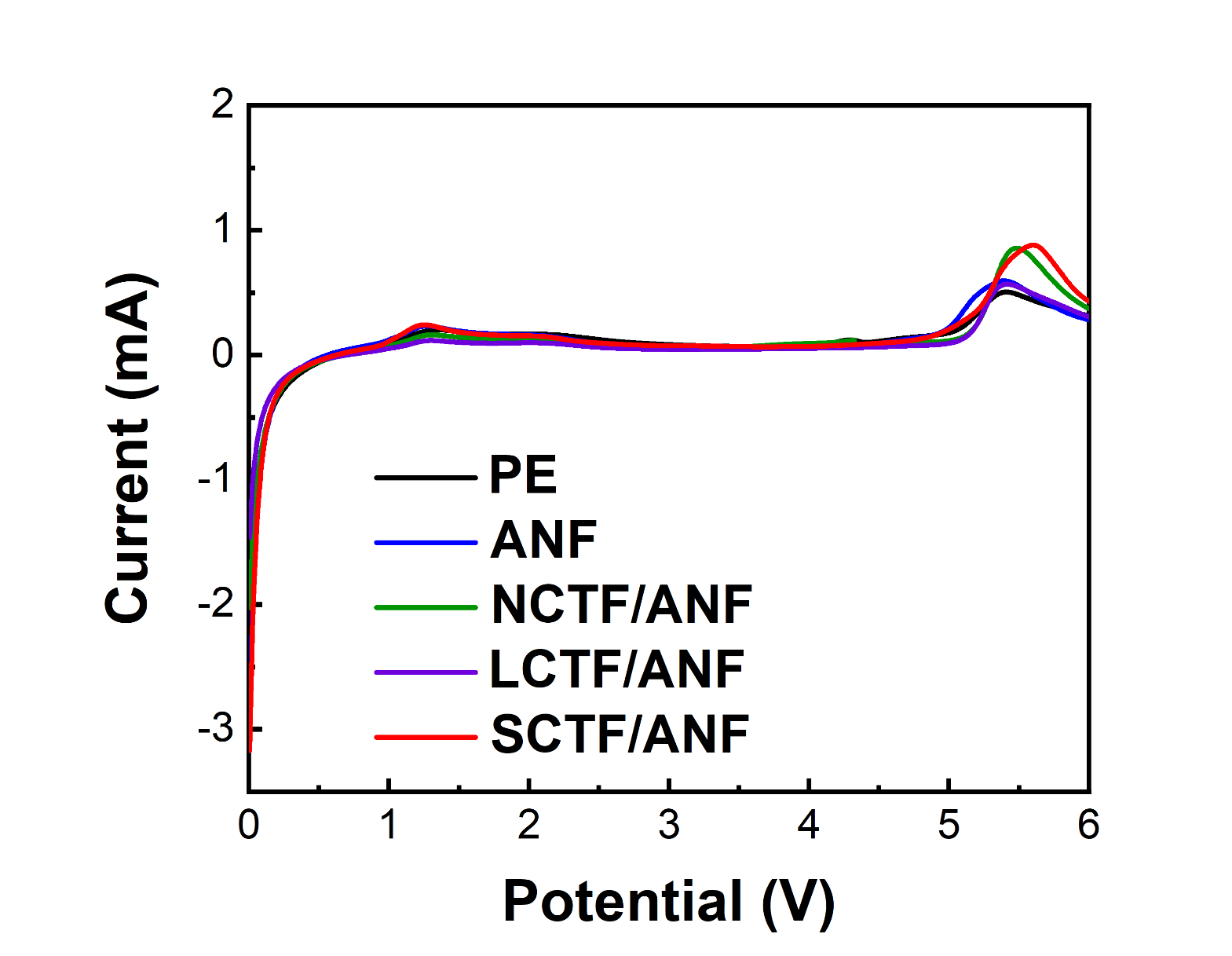


**Figure S16.** LSV curves of the PE, ANF, NCTF/ANF, LCTF/ANF and SCTF/ANF separators.


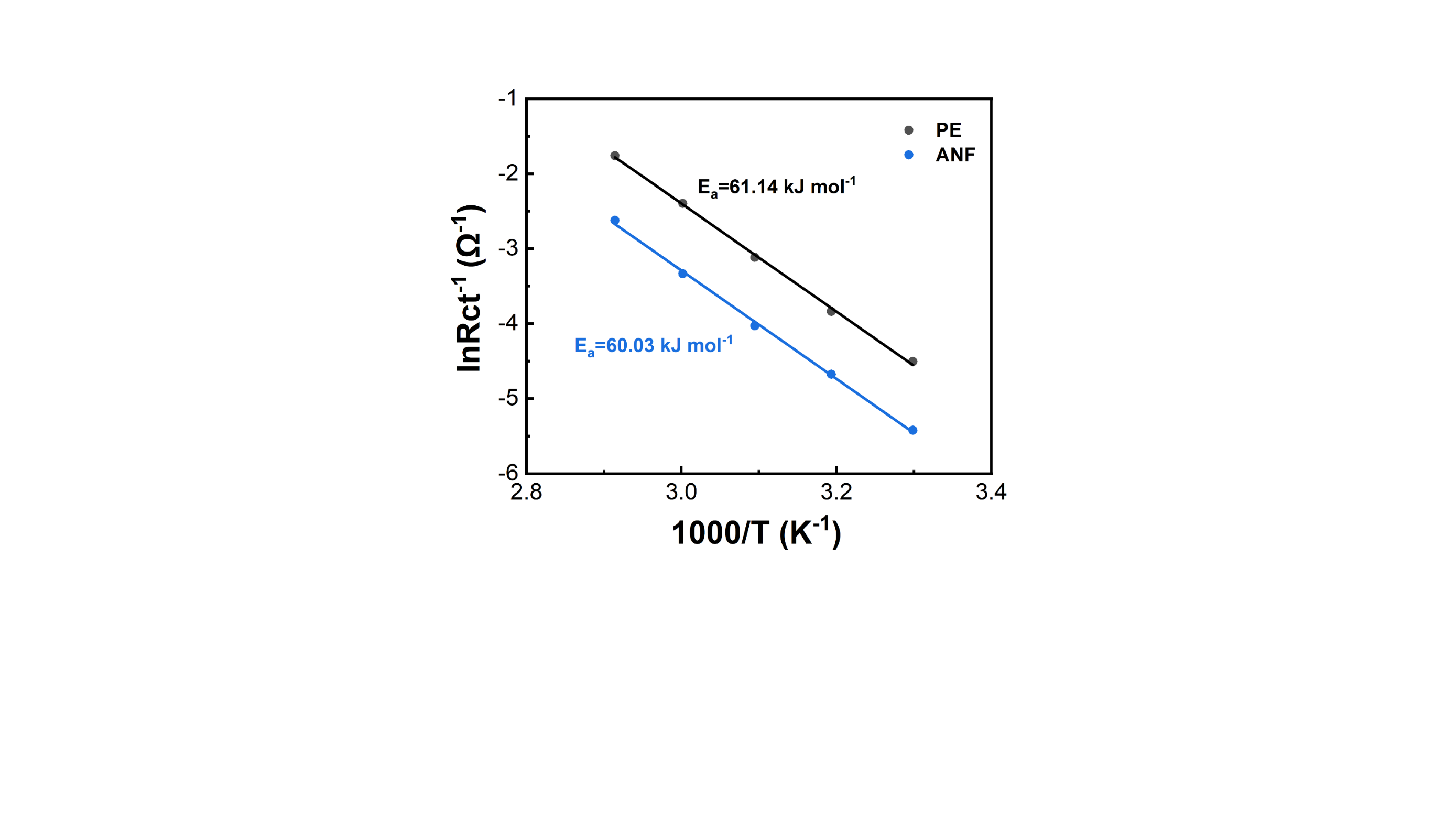


**Figure S17.** The calculation of activation energy ($E_{a}$) from Nyquist plots.


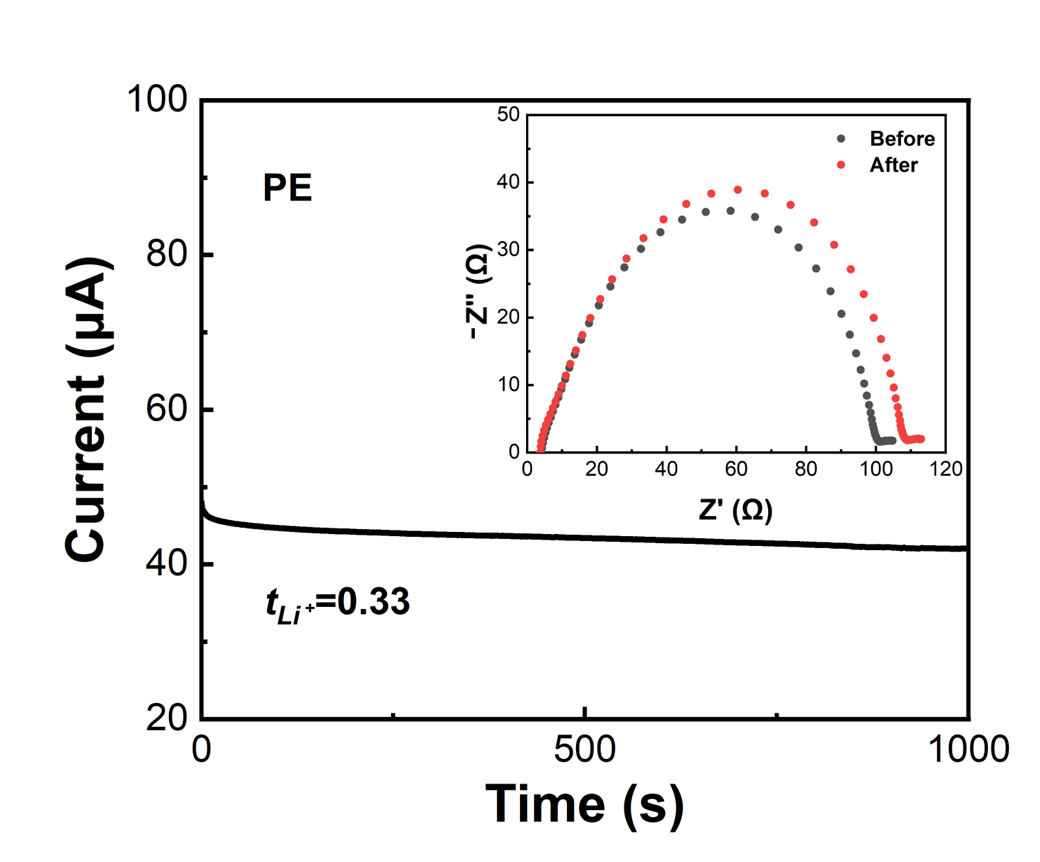


**Figure S18.** Chronoamperometry profiles of PE separator. Inset are the AC impedance spectra before and after polarization.


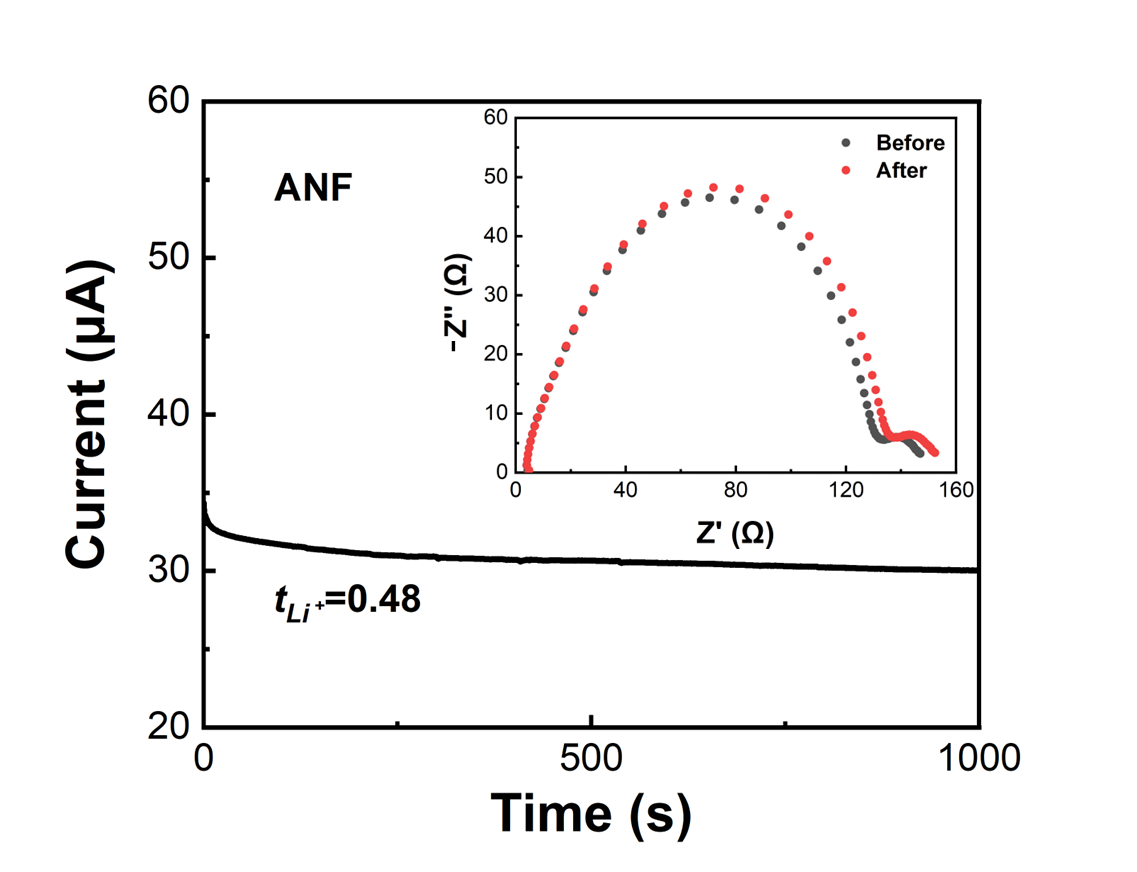


**Figure S19.** Chronoamperometry profiles of ANF separator. Inset are the AC impedance spectra before and after polarization.


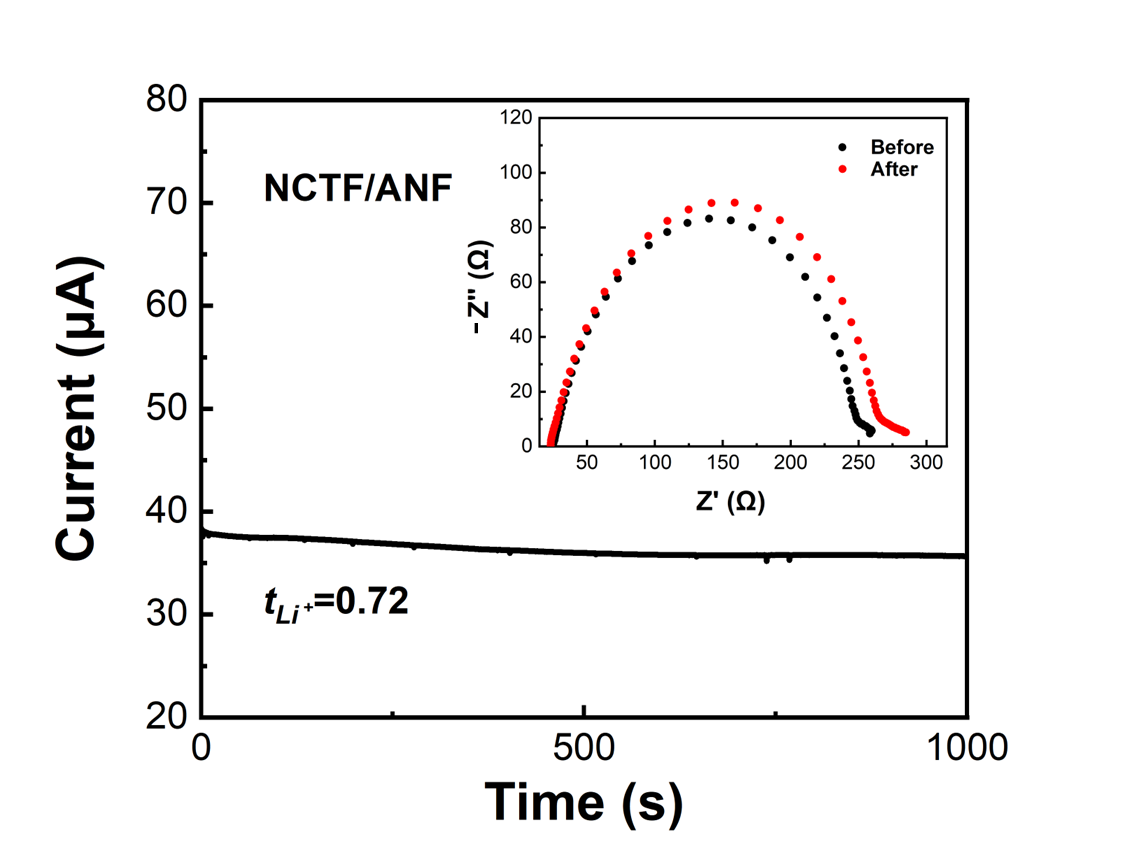


**Figure S20.** Chronoamperometry profiles of NCTF/ANF separator. Inset are the AC impedance spectra before and after polarization.


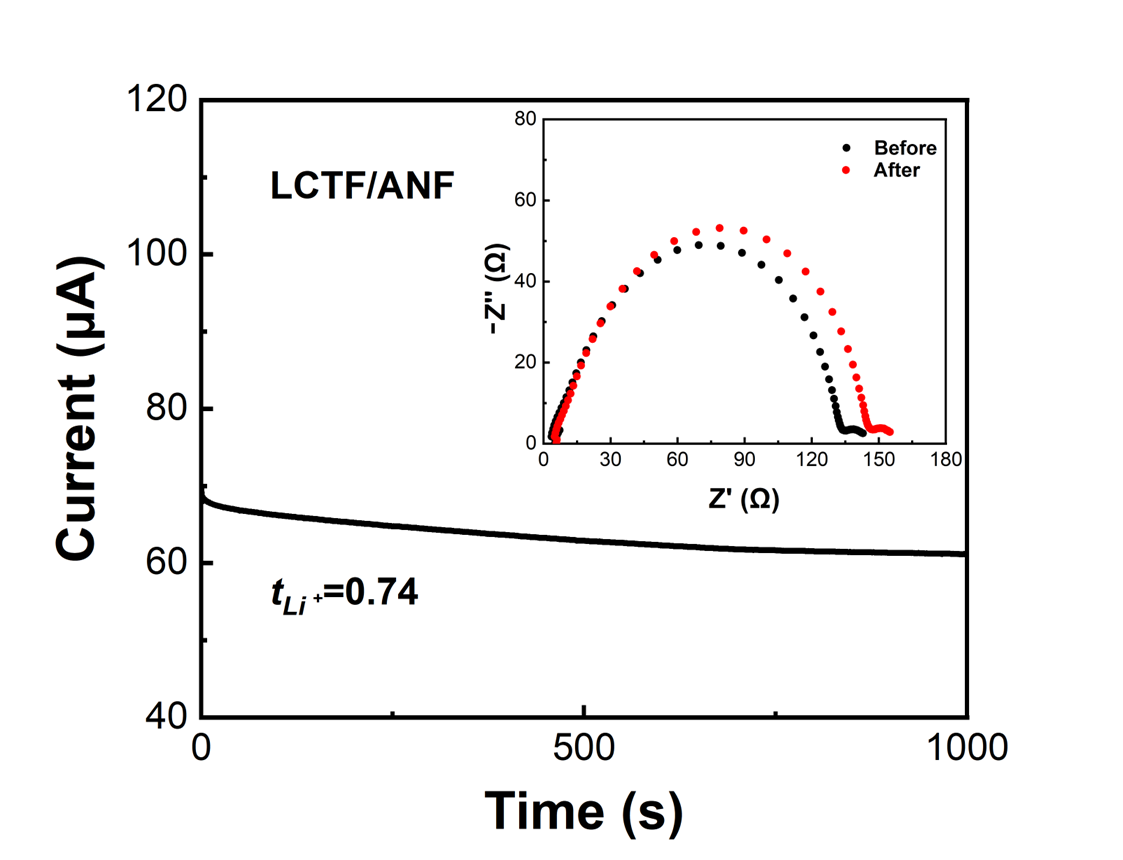


**Figure S21.** Chronoamperometry profiles of LCTF/ANF separator. Inset are the AC impedance spectra before and after polarization.


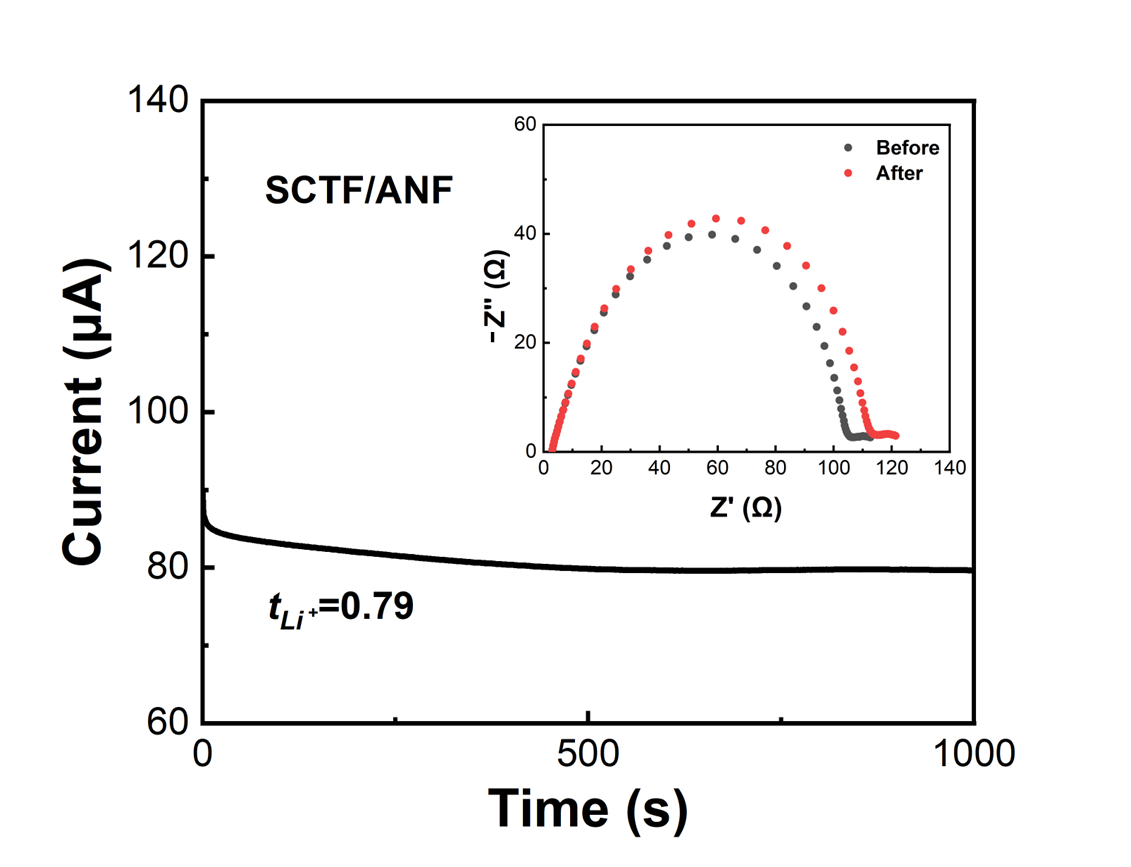


**Figure S22.** Chronoamperometry profiles of SCTF/ANF separators. Inset are the AC impedance spectra before and after polarization.


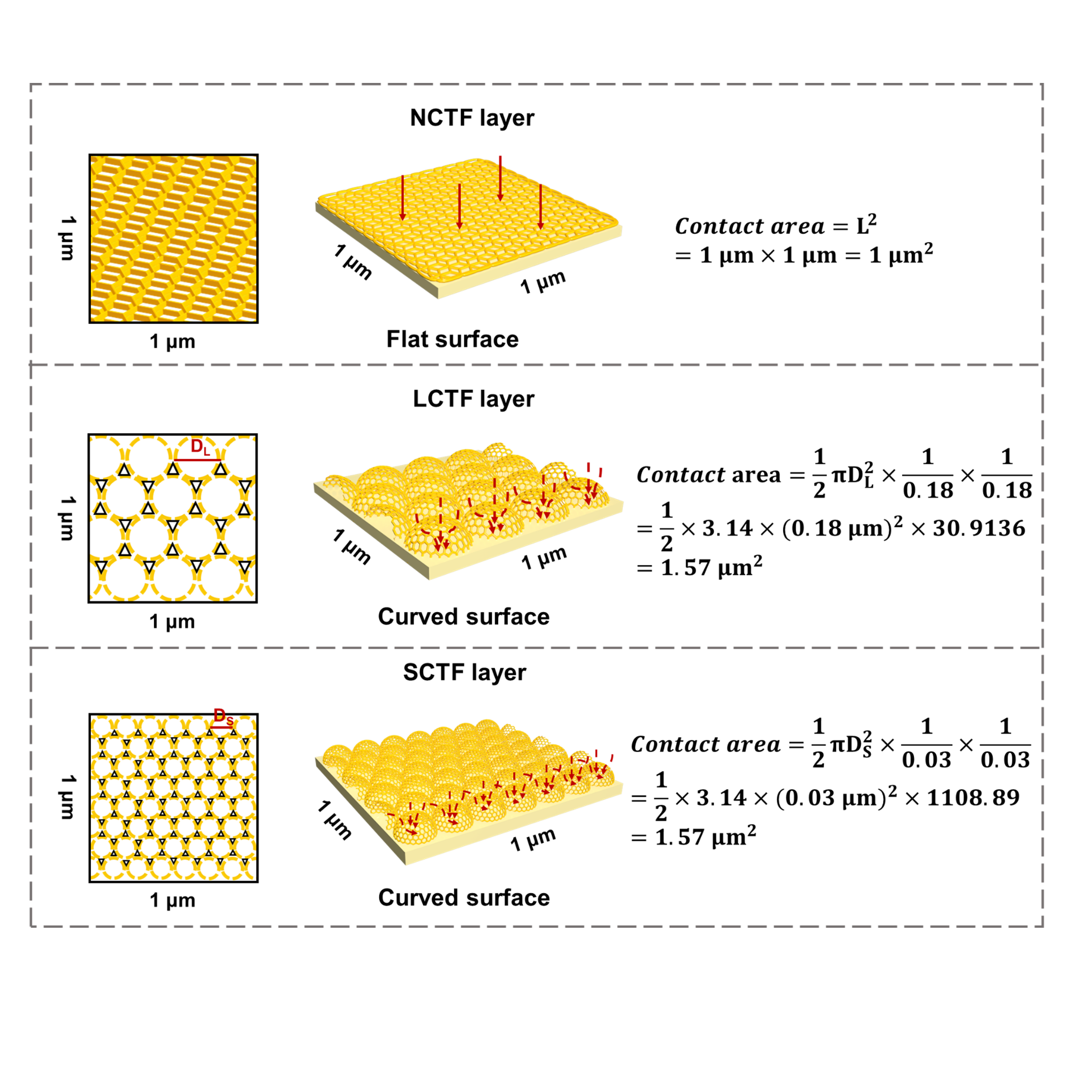


**Figure S23.** The contact area of liquid electrolyte with the flat surface of NCTF layer, the curved surface of LCTF and SCTF layers in area of 1 × 1 μm^2^.

When the liquid electrolyte is exposed to CTF porous layers, the transport behaviors are related to their contact area. In 1 × 1 μm^2^ area, in ideal case, the contact area of flat surface of NCTF layer is calculated by $S_{N}=L^{2}$, and that of the curved surface of LCTF (*S_L_*) and SCTF (*S_s_*) hemispheres are calculated by $S=\frac{1}{2}{\pi D}^{2}N$ (where D is the diameter of the nanosphere, *N* is the number of nanospheres within the 1 × 1 μm^2^ area).


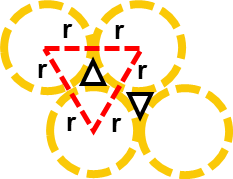


**Figure S24.** Schematic illustration of the pore structure and calculation of the surface area of pore B.

Considering ion transport through pore A and pore B happen at the same time when liquid electrolyte was exposed to CTF porous layers, the comprehensive transport behavior was determined by averaging the contributions of different ion pathways. To do the weighted calculation, the ratio of the surface areas of pore A (●) and pore B (▲) is needed. As shown in **Figure S24**, the area of pore B was equal to area of the equilateral triangle minus the three fragment areas of the circles.

Equilateral triangle area: $\frac{\surd3}{4}{(2r)}^{2}=\surd3r^{2}$

Three fragment areas of the circles: $3\times\frac{60^{\circ}}{360^{\circ}}\times\pi r^{2}=0.5\pi r^{2}$

Therefore, the area of pore B is $0.161r^{2}$, and the pore A-to-pore B ratio of surface area is $20:1$. But according to **Figure S23**, the surface area of the pore A is 1.57 greater than pore B, when its curved, thus making the final pore A-to-pore B ratio of surface area 29.26:1.

According to equation 9, the currents that pass at each stage are calculated by multiplying the current densities with the area.

$$\begin{aligned} I_{{Li}^{+}}^{▲}=j_{{Li}^{+}}^{▲}S_{▲}\#\left( 9 \right) \end{aligned}$$

Where $I_{{Li}^{+}}^{▲}$ is current passing through the pore B, $j_{{Li}^{+}}^{▲}$ is current density from ions passing through pore B, $S_{▲}$ is area of pore B.

The total current for each ion was calculated by adding the two pathways (current passing through pore A, indicated by the circle and current passing through pore B, indicated by the triangle).

$$\begin{aligned} I_{{Li}^{+}}=I_{{Li}^{+}}^{▲}+I_{{Li}^{+}}^{●}\#\left( 10 \right) \end{aligned}$$

$$\begin{aligned} I_{{PF}_{6}^{+}}=I_{{PF}_{6}^{+}}^{▲}+I_{{PF}_{6}^{+}}^{●}\#\left( 11 \right) \end{aligned}$$

Considering the total currents, the Li^+^ transference number can be derived from equation 12.

$$\begin{aligned} t_{{Li}^{+}}=\frac{I_{{Li}^{+}}}{I_{{Li}^{+}}+I_{{PF}_{6}^{+}}}\#\left( 12 \right) \end{aligned}$$

**
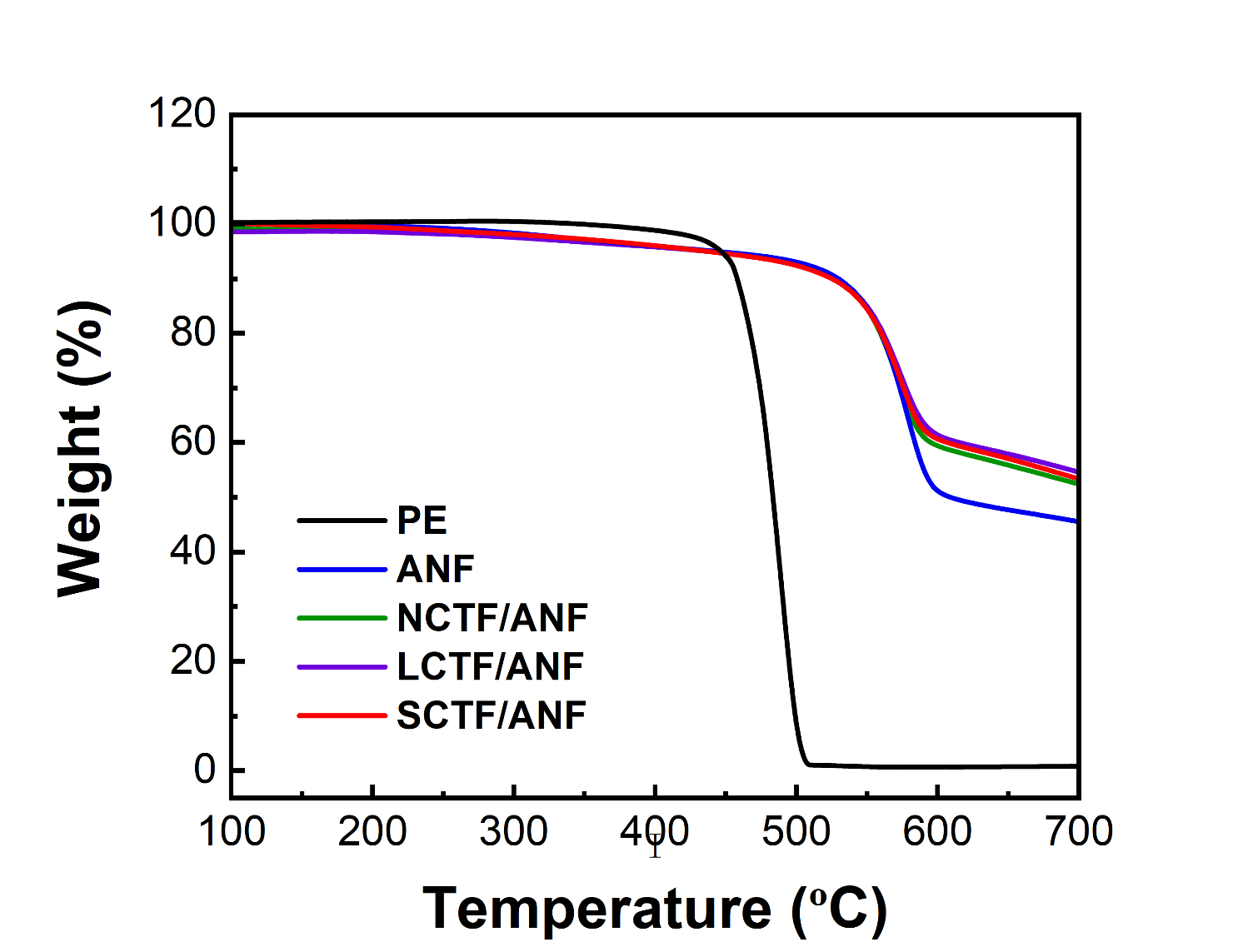
**

**Figure S25.** TGA curves of PE, ANF, NCTF/ANF, LCTF/ANF and SCTF/ANF separators.


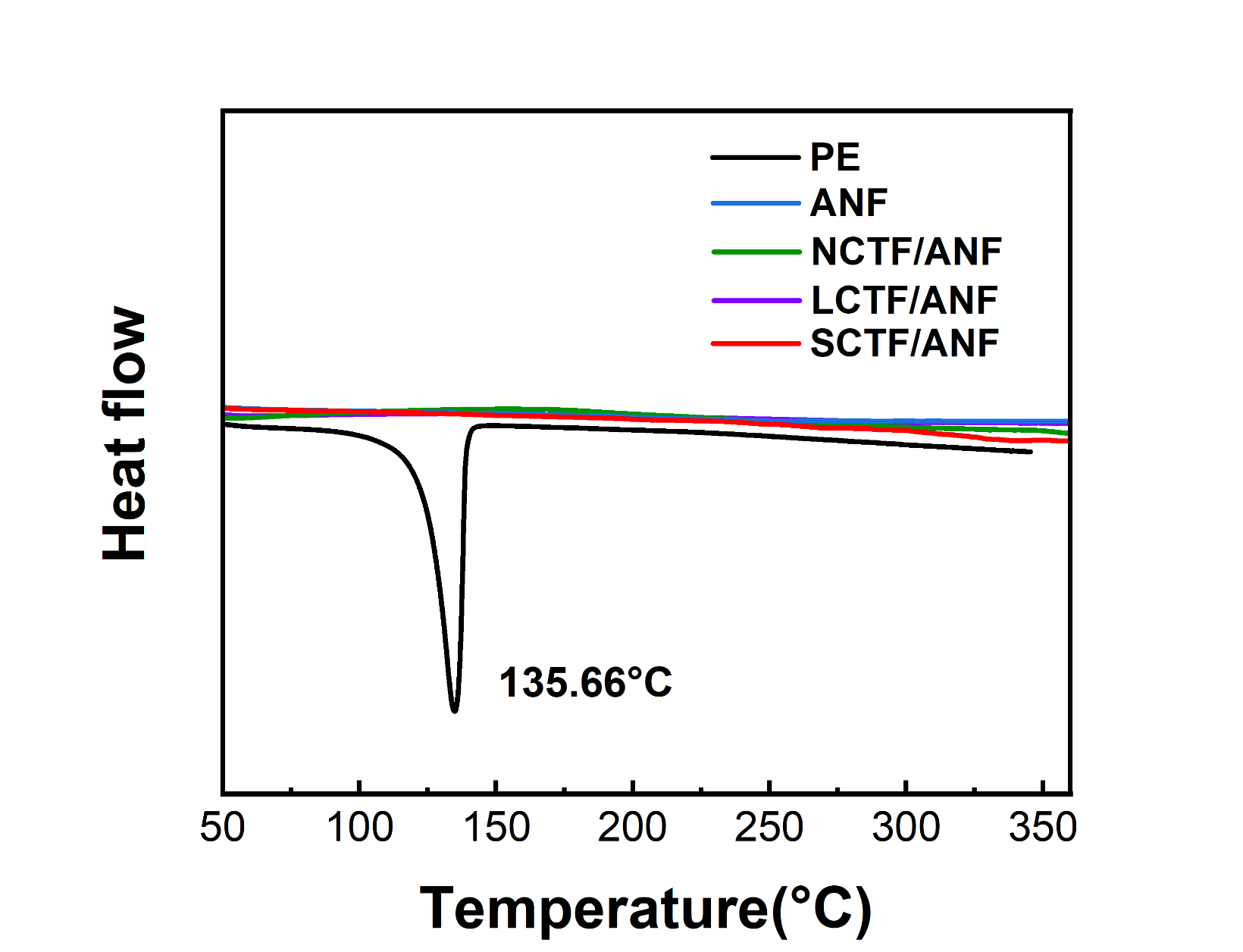


**Figure S26.** DSC curves of PE, ANF, NCTF/ANF, LCTF/ANF and SCTF/ANF separators.


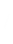
**
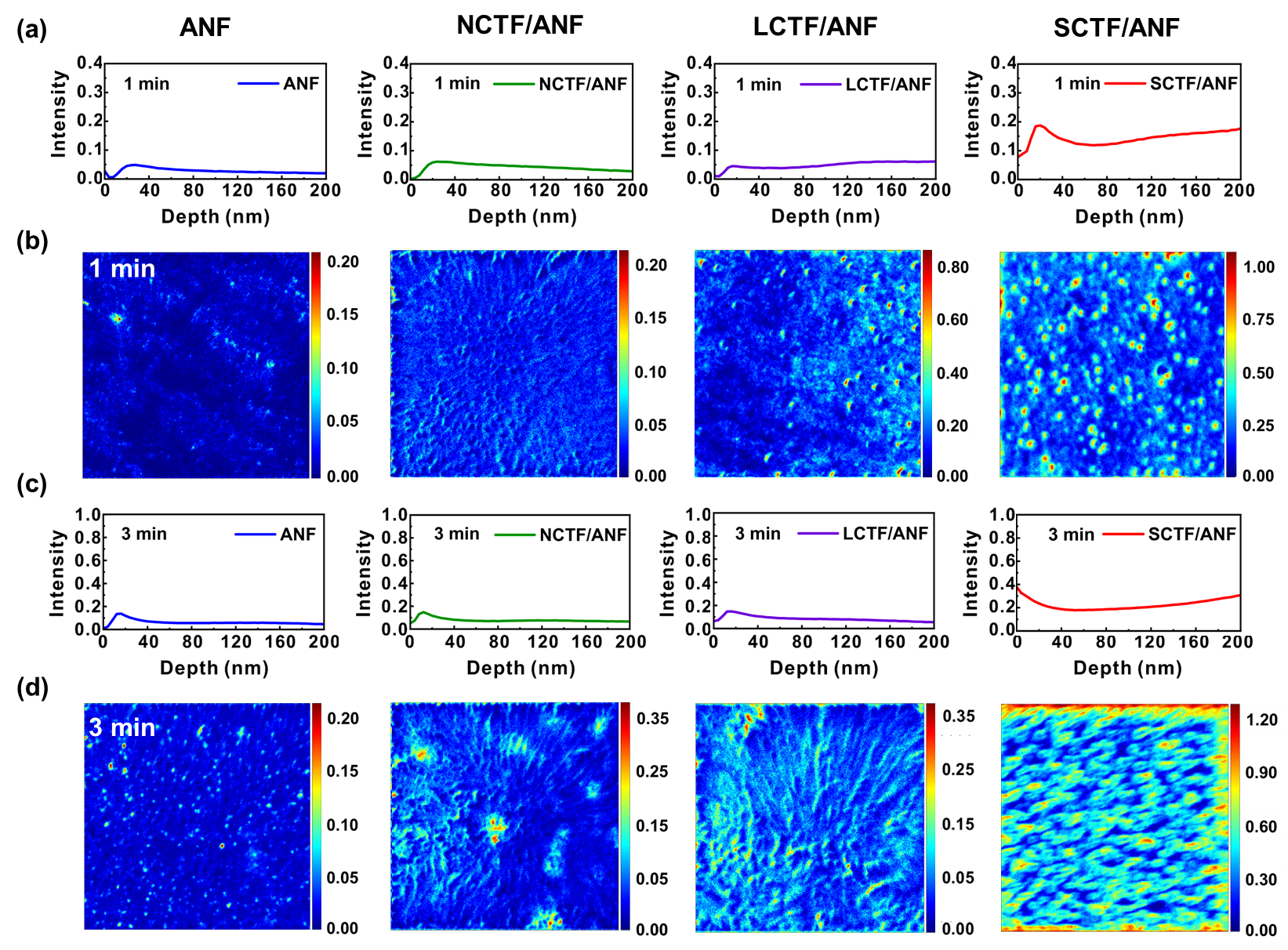
**

**Figure S27** K^+^ concentration and their compiled maps across the ANF, NCTF/ANF, LCTF/ANF and SCTF/ANF separators for the various active times of (a, b) 1 min and (c, d) 3 min by TOF-SIMS depth profiling. (b, d) Plot of K^+^ concentration versus depth. (c, e) Top-view images of K^+^ concentration. The unit of intensity is counts per extraction (cts/ex). One extraction corresponds to one pulse of the TOF or equivalently, one pixel in the FIB scan.


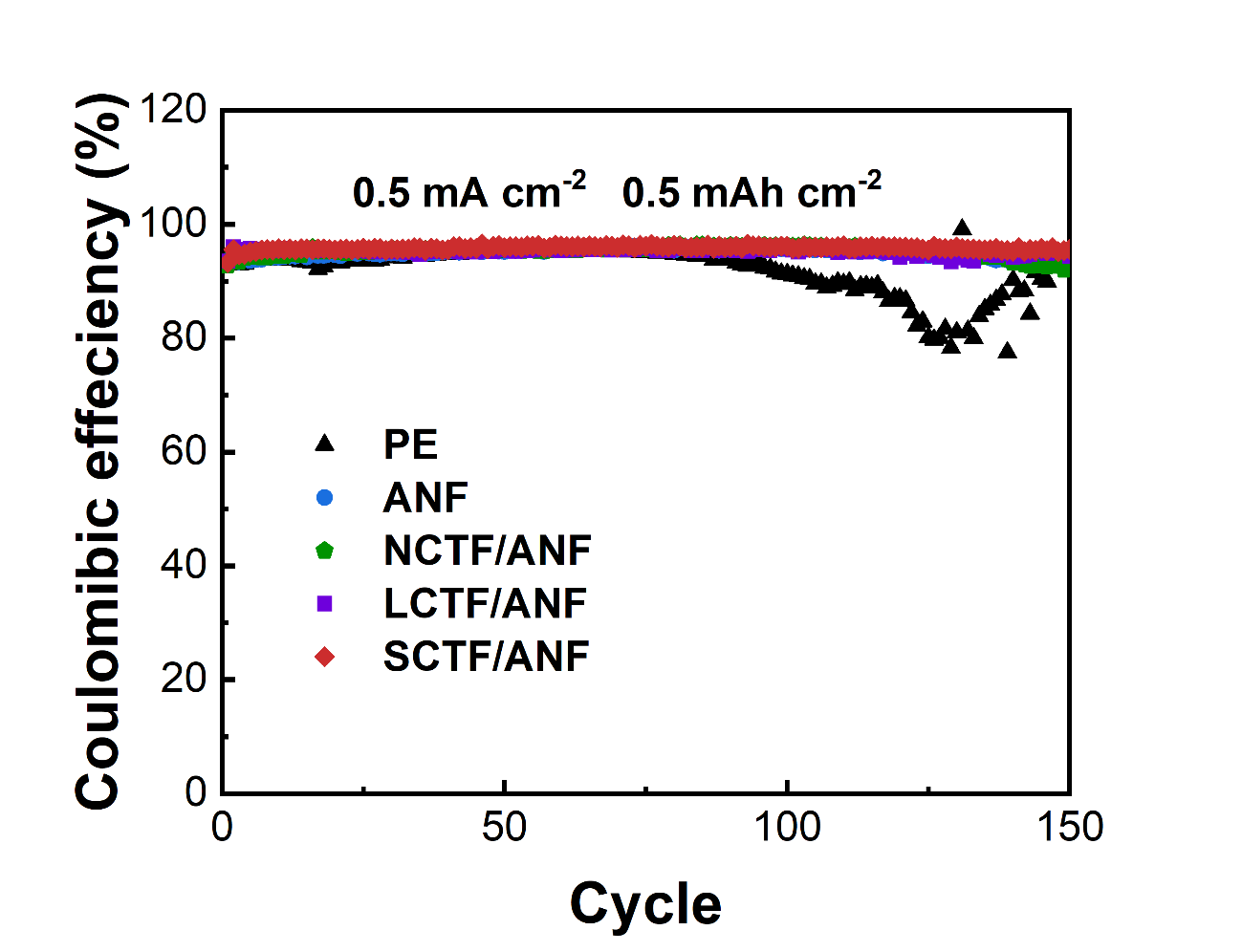


**Figure S28** Coulombic efficiency (CE) at 0.5 mA cm^-2^ with the capacity of 0.5 mAh cm^-2^.

**
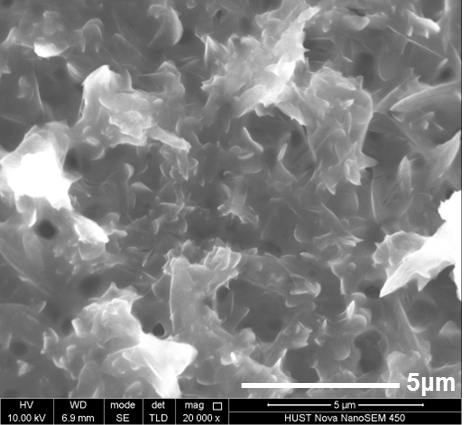
**

**Figure S29.** FE-SEM images of Li metal from Li//Li cells assembled with PE separator after Li plating at a current density of 1 mA cm^-2^.


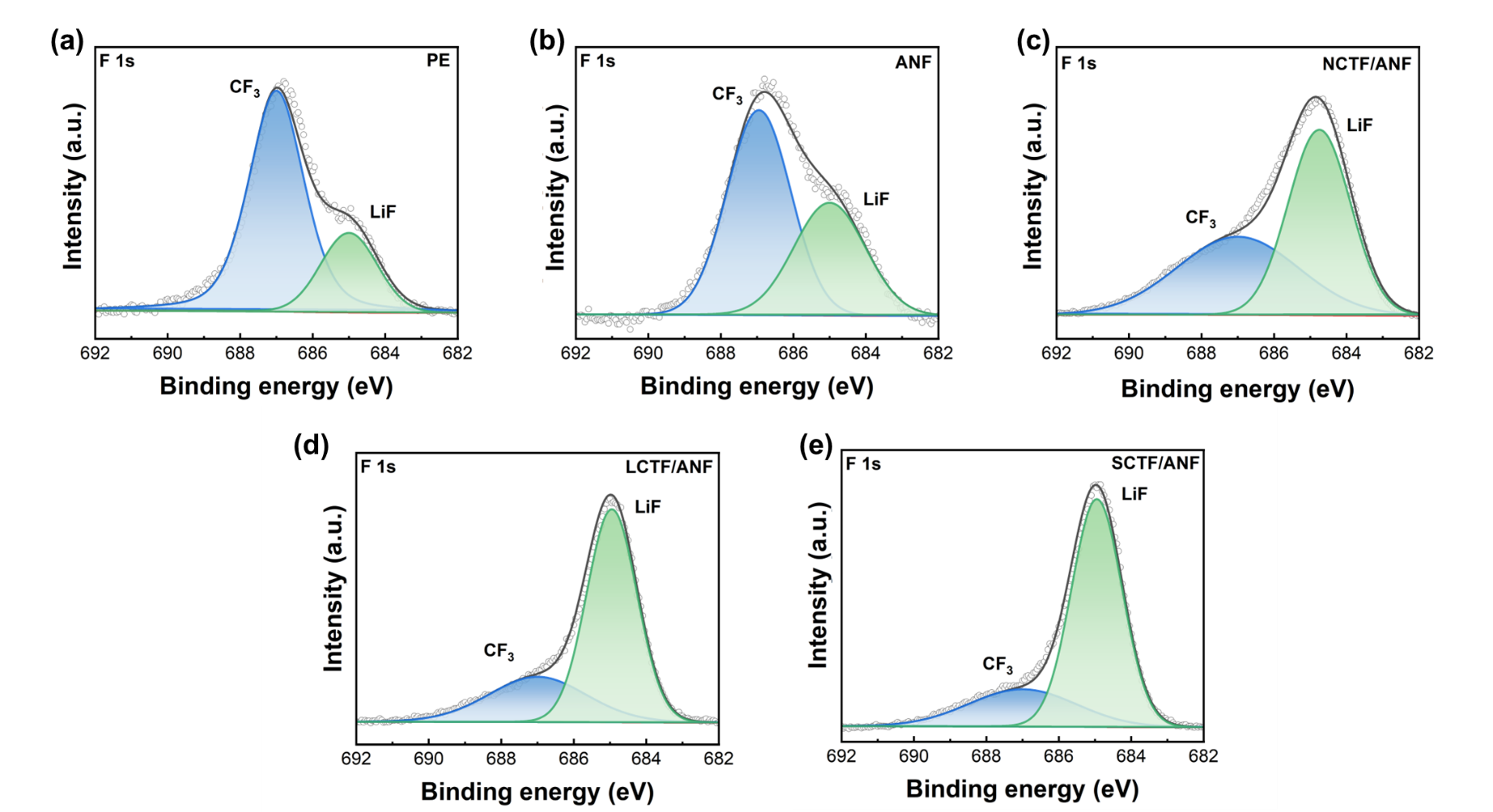


**Figure S30** XPS spectra of SEI layers in Li//Li symmetric cells with (a) PE, (b) ANF, (c) NCTF/ANF, (d) LCTF/ANF and (e) SCTF/ANF separators at a current density of 1 mA cm^-2^ with an area capacity of 1 mAh cm^-2^ after 100 h.


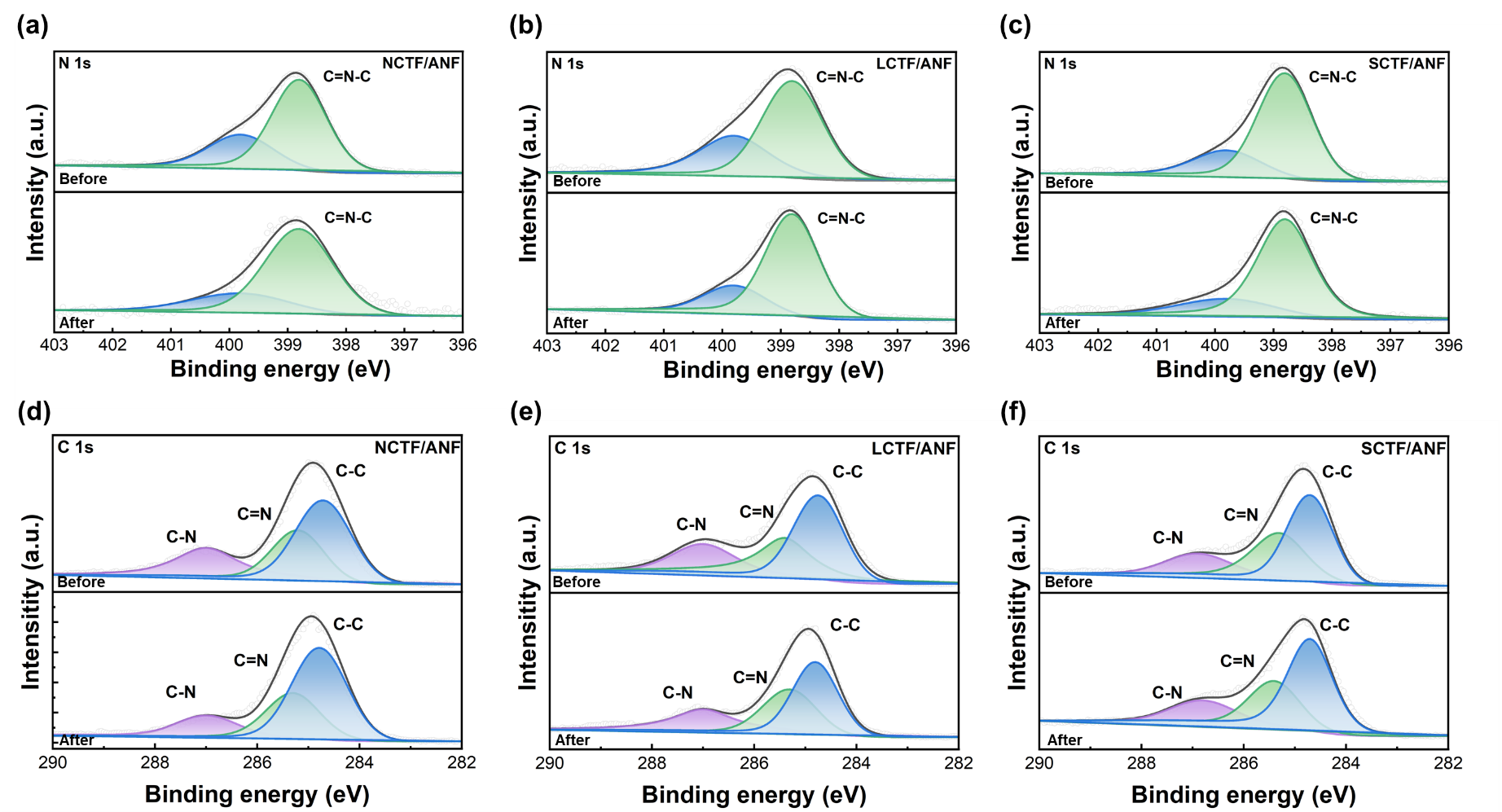


**Figure S31** (a-c) N 1s and (d-f) C 1s XPS spectra for NCTF/ANF, LCTF/ANF and SCTF/ANF separators in Li//Li symmetric cells before and after 100 h at a current density of 1 mA cm^-2^ with an area capacity of 1 mAh cm^-2^.


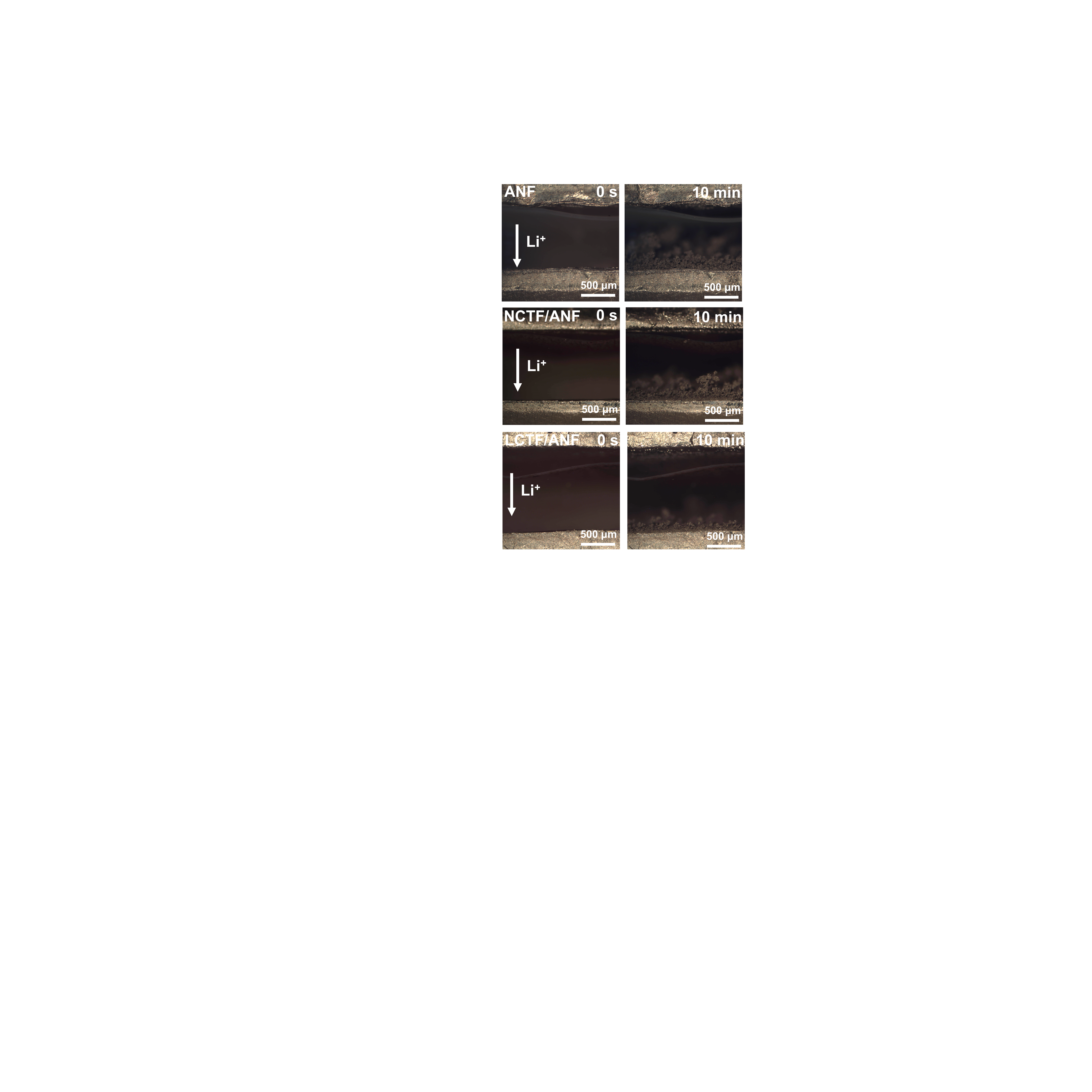


**Figure S32.** Morphology of lithium in Li//Li symmetrical cells with ANF, NCTF/ANF and LCTF/ANF separators after discharging for 10 min at 3 mA cm^-2^ using in situ optical microscopy.


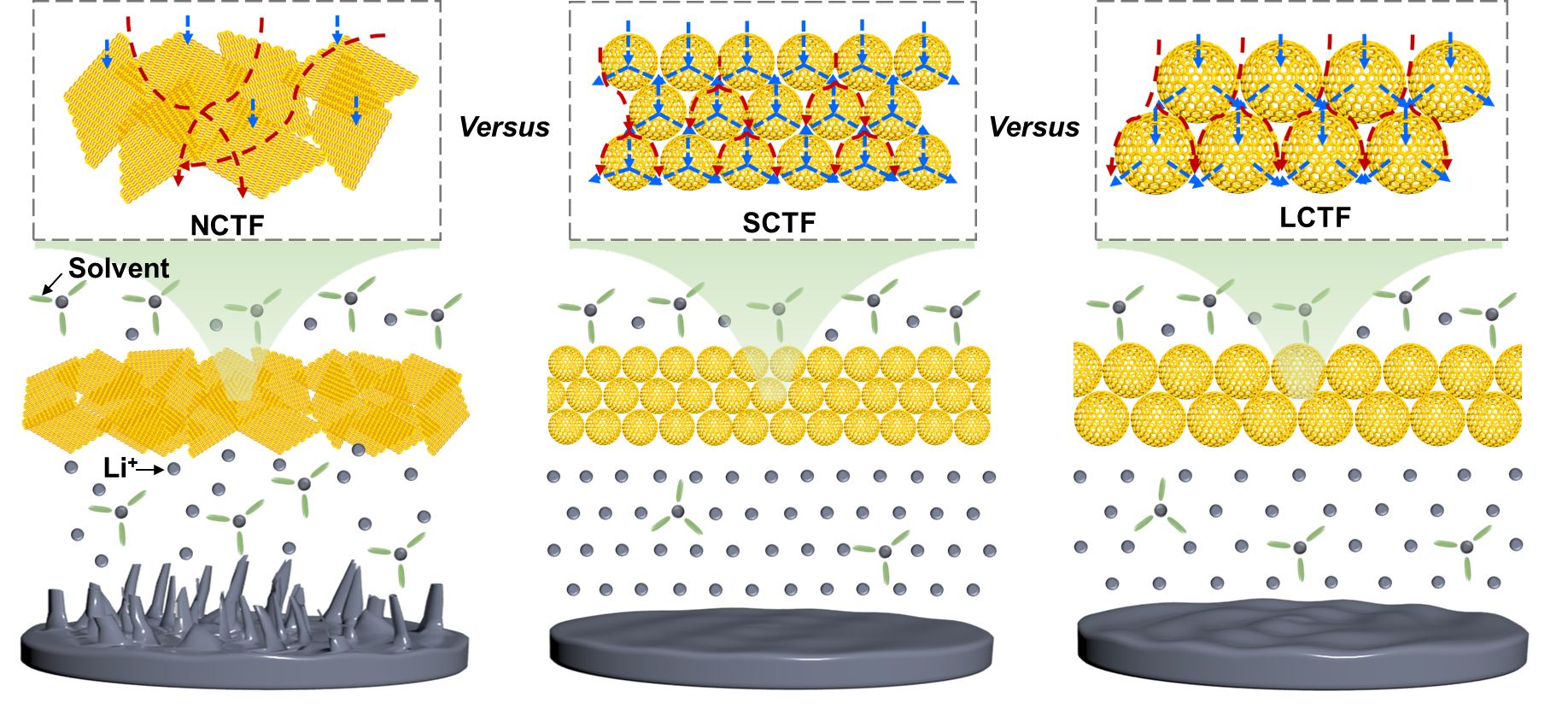


**Figure S33** Schematic illustration of the ion flow in NCTF, LCTF and SCTF coating layers of different separators. The shortened and interpenetrative ion pathway in SCTF coating layer leads to fast ion transport and uniform ion distribution.


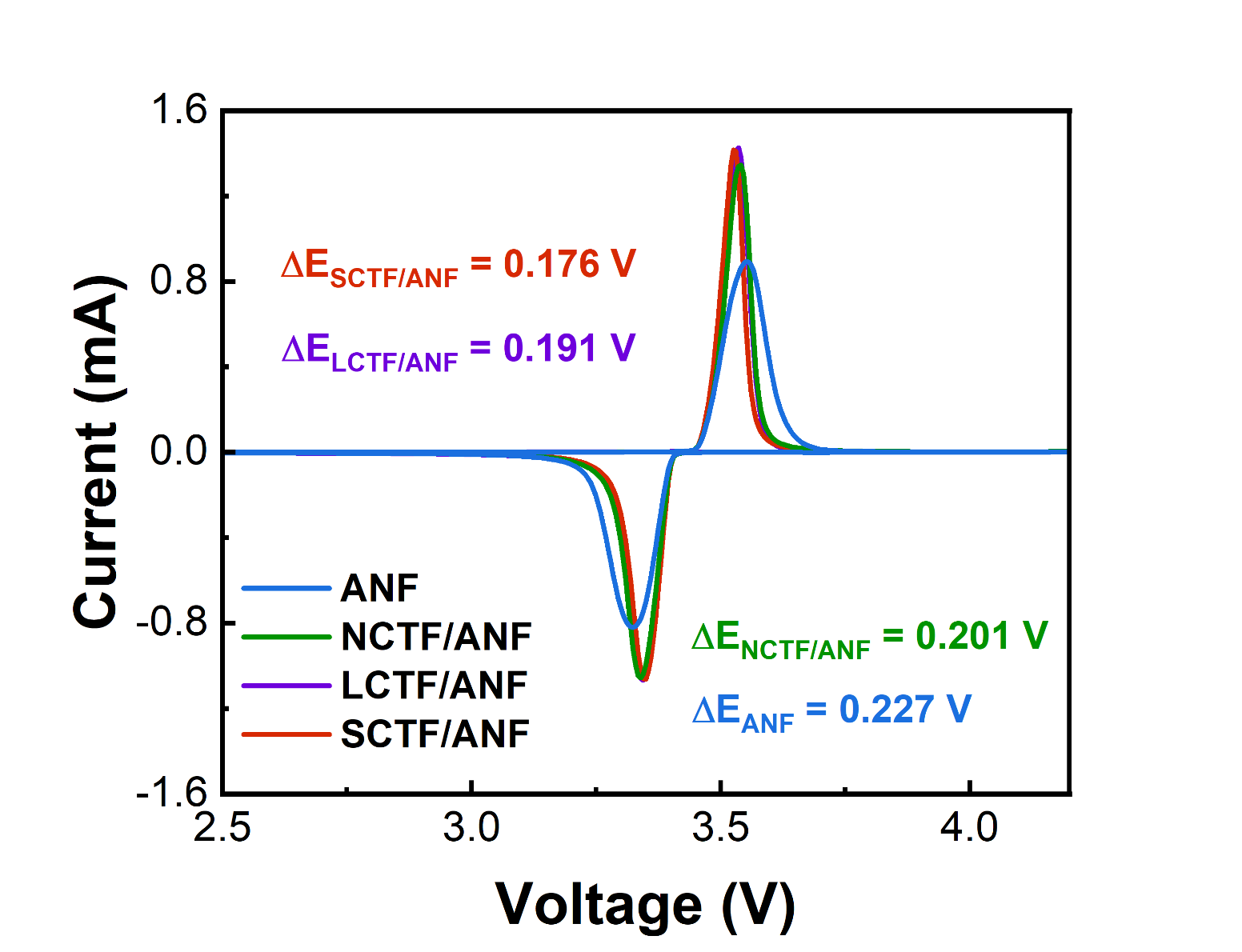


**Figure S34** The CV profiles of Li//LFP cells were tested between 2.5 and 4.2 V with a scan rate of 0.1 mV s^-1^.


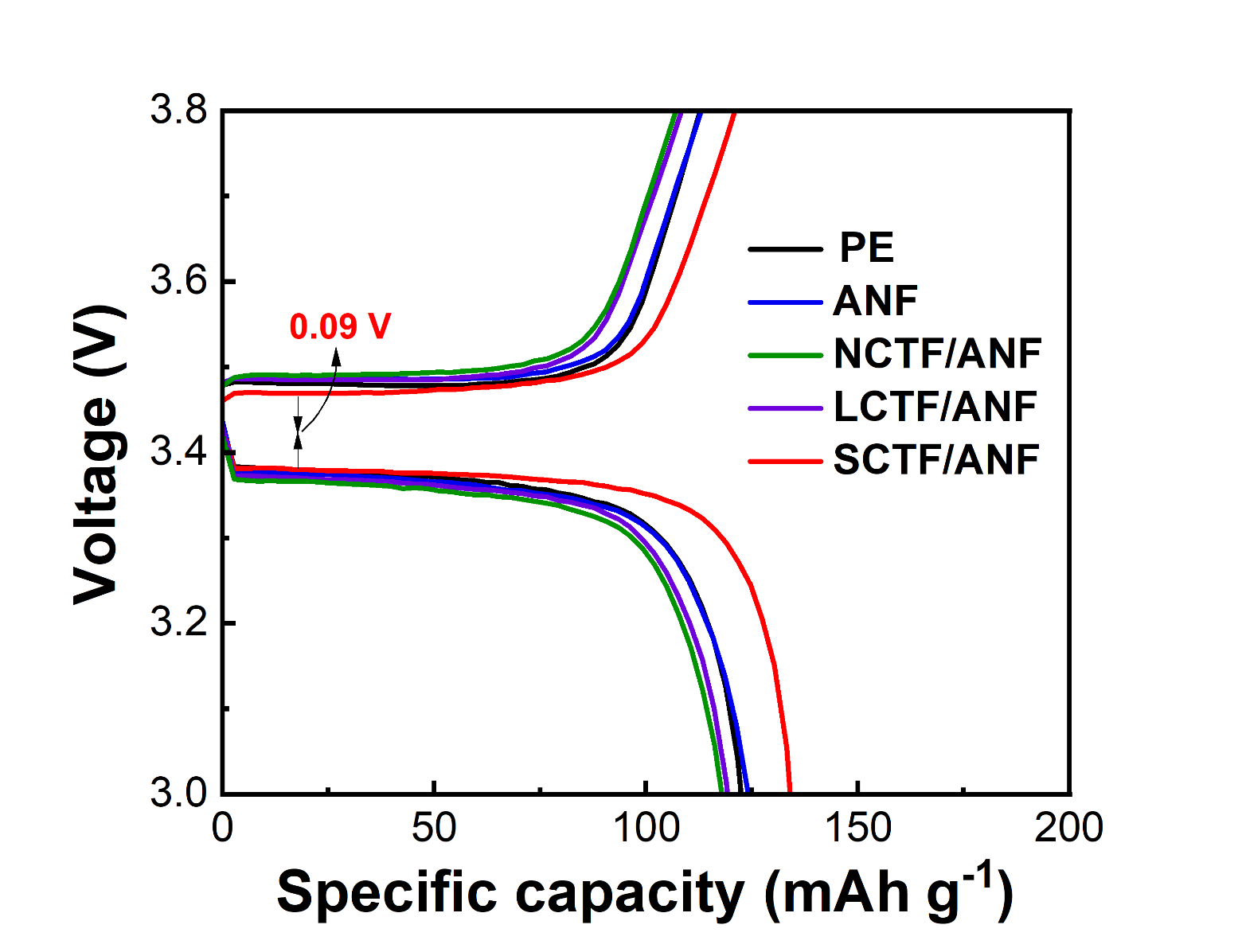


**Figure S35.** The discharge/charge curves of different separators assembled cells at 1 C.

**Supplementary Tables**

**Table S1** The values of the BET surface area, pore volume, porosity and tortuosity of the ANF, NCTF/ANF, LCTF/ANF and SCTF/ANF separators.

| **Sample** | **Surface area (m^2^/g)** | **Pore volume (cm^3^/g)** | **Porosity**  **(%)** | **Tortuosity**  **(τ)** |
| --- | --- | --- | --- | --- |
| **ANF** | 264.13 | 1.70 | 52.7 | 1.58 |
| **NCTF/ANF** | 223.73 | 1.50 | 51.1 | 1.64 |
| **LCTF/ANF** | 198.06 | 1.09 | 51.7 | 1.60 |
| **SCTF/ANF** | 213.14 | 1.40 | 52.5 | 1.58 |

**Table S2** The values of the area of the stainless electrode, and bulk resistance for ionic conductivity calculation.

| **Sample** | **Electrode area**  **(cm^2^)** | **R_0_**  **(Ω)** | **Ionic conductivity**  **σ (mS/cm)** |
| --- | --- | --- | --- |
| PE | 1.96 | 2.5 | 0.19 |
| ANF | 1.96 | 1.95 | 1.05 |
| NCTF/ANF | 1.96 | 1.88 | 1.11 |
| LCTF/ANF | 1.96 | 1.65 | 1.27 |
| SCTF/ANF | 1.96 | 1.49 | 1.41 |

**Table S3** Calculated current and current density of ions transporting through or along the two-layered and three-layered CTF from MD simulation.

|  | **Pore A**  **(Two-layered CTF)** | **Pore A**  **(Three-layered CTF)** | **Pore B_1_**  **(small pore model)** | **Pore B_2_**  **(big pore model)** |
| --- | --- | --- | --- | --- |
| $I_{{Li}^{+}}$  (nA) | 0.32 | 0.21 | 0.12 | 0.12 |
| $j_{{Li}^{+}}$  (pA/nm^2^) | 1.12 | 0.72 | 7.02 | 6.82 |
| $I_{{PF}_{6}^{-}}$  (nA) | -0.04 | -0.07 | -0.23 | -0.25 |
| $j_{{PF}_{6}^{-}}$  (pA/nm^2^) | -0.14 | -0.23 | -13.18 | -14.24 |

Note: Current (*I*) = Charge/Time; Current Density (*j*) = Current (*I*)/Area (A); Ionic charge =1.6 × 10 ^-19^ C

**Table S4** Current densities of ions through multiple CTF layers (pore A with different thicknesses) and $t_{{Li}^{+}}$.

| **Number of CTF Layers** | **Li^+^ Current Density (pA/nm^2^)** | **PF_6_^-^ Current Density**  **(pA/nm^2^)** | **Li^+^ transference number** |
| --- | --- | --- | --- |
| 1 | 1.79 | -1.27 | 0.58 |
| 2 | 1.11 | -0.137 | 0.89 |
| 3 | 0.72 | -0.232 | 0.76 |
| 4 | 0.36 | -0.235 | 0.61 |
| 5 | 0.098 | -0.084 | 0.54 |
| 6 | 0.084 | -0.072 | 0.52 |
| 7 | 0.042 | -0.072 | 0.35 |
| 8 | 0.025 | -0.108 | 0.18 |

**Table S5** Calculated currents of ions passing through different CTF systems and $t_{{Li}^{+}}$.

|  | $I_{{Li}^{+}}^{●}$  (pA) | $I_{{Li}^{+}}^{▲}$  (pA) | $I_{{Li}^{+}}$  (pA) | $I_{{PF}_{6}^{-}}^{●}$  (pA) | $I_{{PF}_{6}^{-}}^{▲}$  (pA) | $I_{{PF}_{6}^{-}}$  (pA) | $t_{{Li}^{+}}$ |
| --- | --- | --- | --- | --- | --- | --- | --- |
| Pore B_1_  Three-layered CTF | 0.212 | 0.070 | 0.282 | -0.068 | -0.132 | -0.200 | 0.59 |
| Pore B_1_  Two-layered CTF | 0.322 | 0.070 | 0.392 | -0.040 | -0.132 | -0.172 | 0.70 |
| Pore B_2_  Three-layered CTF | 0.212 | 0.068 | 0.280 | -0.068 | -0.145 | -0.213 | 0.57 |
| Pore B_2_  Two-layered CTF | 0.322 | 0.068 | 0.390 | -0.040 | -0.145 | -0.185 | 0.68 |

**Table S6** Comparison of the performances of SCTF/ANF separator with commercial polyolefin separators.

| **Sample** | **Porosity**  **(%)** | **Electrolyte uptakes**  **(%)** | **Ionic conductivity**  **(mS cm^-1^)** | **Li^+^ transference number** | **Thermal shrinkages**  **（150℃）** |
| --- | --- | --- | --- | --- | --- |
| PE  (Hipore) | 40 | 105 | 0.5 | 0.405 | ~ 30% |
| PP/PE/PP  (Celgard 2325) | 41 | 110 | 0.528 | 0.287 | ~ 20% |
| PP  (Celgard 2500) | 39 | 100 | 0.546 | 0.438 | ~ 25% |
| SCTF/ANF | 52 | 160 | 1.41 | 0.79 | 0% |

**Supplementary References**

[1] Y. Huang, J. E. Pemberton, *Colloids Surf. Physicochem. Eng. Asp.* **2010**, *360*, 175.

[2] M. Di, X. Sun, L. Hu, L. Gao, J. Liu, X. Yan, X. Wu, X. Jiang, G. He, *Adv. Funct. Mater.* **2022**, *32*, 2111594.

[3] N. Wang, G. Cheng, L. Guo, B. Tan, S. Jin, *Adv. Funct. Mater.* **2019**, *29*, 1904781.

[4] R. Sun, B. Tan, *Chem. – Eur. J.* **2023**, *29*, e202203077.

[5] H. Pei, C. Yang, Q. Wu, X. Zhou, X. Xie, B. Hwang, Y. Ye, *J. Mater. Chem. A* **2022**, *10*, 5317.

[6] W. Sun, Q. M. Li, P. Xiao, P. Carbone, *J. Energy Storage* **2024**, *97*, 112940.

[7] B. R. Brooks, C. L. Brooks, A. D. Mackerell, L. Nilsson, R. J. Petrella, B. Roux, Y. Won, G. Archontis, C. Bartels, S. Boresch, A. Caflisch, L. Caves, Q. Cui, A. R. Dinner, M. Feig, S. Fischer, J. Gao, M. Hodoscek, W. Im, K. Kuczera, T. Lazaridis, J. Ma, V. Ovchinnikov, E. Paci, R. W. Pastor, C. B. Post, J. Z. Pu, M. Schaefer, B. Tidor, R. M. Venable, H. L. Woodcock, X. Wu, W. Yang, D. M. York, M. Karplus, *J. Comput. Chem.* **2009**, *30*, 1545.

[8] K. Vanommeslaeghe, A. D. Jr. MacKerell, *J. Chem. Inf. Model.* **2012**, *52*, 3144.

[9] K. Vanommeslaeghe, E. P. Raman, A. D. Jr. MacKerell, *J. Chem. Inf. Model.* **2012**, *52*, 3155.

[10] J. C. Phillips, D. J. Hardy, J. D. C. Maia, J. E. Stone, J. V. Ribeiro, R. C. Bernardi, R. Buch, G. Fiorin, J. Hénin, W. Jiang, R. McGreevy, M. C. R. Melo, B. K. Radak, R. D. Skeel, A. Singharoy, Y. Wang, B. Roux, A. Aksimentiev, Z. Luthey-Schulten, L. V. Kalé, K. Schulten, C. Chipot, E. Tajkhorshid, *J. Chem. Phys.* **2020**, *153*, 044130.

[11] T. Darden, D. York, L. Pedersen, *J. Chem. Phys.* **1993**, *98*, 10089.

[12] W. Humphrey, A. Dalke, K. Schulten, *J. Mol. Graph.* **1996**, *14*, 33.
